# Interrogating the protein interactomes of RAS isoforms identifies PIP5K1A as a KRAS-specific vulnerability

**DOI:** 10.1038/s41467-018-05692-6

**Published:** 2018-09-07

**Authors:** Hema Adhikari, Christopher M. Counter

**Affiliations:** 1 Department of Pharmacology and Cancer Biology, Duke University Medical Center, DUMC-3813, Durham, NC 27713 USA; 2 Department of Radiation Oncology, Duke University Medical Center, DUMC-3813, Durham, NC 27713 USA

## Abstract

In human cancers, oncogenic mutations commonly occur in the *RAS* genes *KRAS*, *NRAS*, or *HRAS*, but there are no clinical RAS inhibitors. Mutations are more prevalent in *KRAS*, possibly suggesting a unique oncogenic activity mediated by KRAS-specific interaction partners, which might be targeted. Here, we determine the specific protein interactomes of each RAS isoform by BirA proximity-dependent biotin identification. The combined interactomes are screened by CRISPR-Cas9 loss-of-function assays for proteins required for oncogenic KRAS-dependent, NRAS-dependent, or HRAS-dependent proliferation and censored for druggable proteins. Using this strategy, we identify phosphatidylinositol phosphate kinase PIP5K1A as a KRAS-specific interactor and show that PIP5K1A binds to a unique region in KRAS. Furthermore, PIP5K1A depletion specifically reduces oncogenic KRAS signaling and proliferation, and sensitizes pancreatic cancer cell lines to a MAPK inhibitor. These results suggest PIP5K1A as a potential target in KRAS signaling for the treatment of *KRAS*-mutant cancers.

## Abstract (web summary)

RAS isoforms are frequently mutated in cancer but their inhibition remains challenging. By comparing the protein interactomes of the highly similar isoforms HRAS, NRAS and KRAS, the authors here identify PIP5K1A as a KRAS-specific interactor and a target to inhibit KRAS-driven cell growth.

## Introduction

The family of human RAS small GTPases is comprised of the three genes *KRAS*, *NRAS*, and *HRAS*, which encode the proteins KRAS-4A and KRAS-4B derived from different splicing events (collectively termed KRAS for ease of discussion), NRAS, and HRAS^1^. Oncogenic activating mutations, almost exclusively at G12, G13, or Q61, are commonly found in human cancers^2^. A wealth of experimentation from a wide range of settings indicates that oncogenic mutations in *RAS* genes can initiate, maintain, and promote tumorigenesis^3,4^. Genetic studies also categorically demonstrate that the loss of oncogenic RAS expression in established tumors is antineoplastic^4,5^. For a variety of reasons, however, there are no clinical therapeutics to directly inhibit oncogenic RAS^6^. As such, novel approaches are needed to target these very common oncoproteins.

RAS proteins are nearly identical at the sequence level^1^ and are regulated by, and signal through, many of the same proteins^3,7–10^. Genetic experiments also reveal a high degree of functional redundancy between murine *Hras* and *Kras* genes^11^. Oncogenic mutations in all three *RAS* genes promote tumorigenic growth of human cell lines^12^ and cancer in mice^13–18^. Despite this apparent functional uniformity, ectopic or endogenous expression of oncogenic versions of different Ras isoforms yields variations in severity or types of resulting cancers in murine models^16–21^. In humans, the frequency that the three *RAS* genes are mutated varies extensively. *KRAS* is mutated in nearly a quarter of a wide spectrum of cancers, *NRAS* is mutated in <10% of cancers, but at a high frequency in specific cancers, while *HRAS* is rarely mutated in any cancer^2–4,6^. Thus, experimental as well as epidemiological evidence suggests that there are indeed differences between RAS isoforms.

Some of the above variability may be ascribed to fairly extensive differences in the nucleotide sequence of the three *RAS* genes, which affect the mRNA levels and the rate of translation and correspondingly protein levels^22,23^. Variation in the amino acid sequence, however small, may similarly impart differences in oncogenic activity to RAS isoforms. In this regard, RAS proteins primarily differ in their last ~23 amino acids, a region termed the hypervariable region (HVR)^1,2,21^. The HVR imparts unique post-translational modifications and subcellular localizations^24^ to each isoform, and as such has been argued to underlie, at least in part, isoform differences in oncogenic activity^21^. As RAS isoforms are signaling proteins, differences in their oncogenic activity may manifest in the composition of their protein interactomes. The protein interactomes of oncogenic KRAS^25^ and HRAS^26^ have previously been characterized by immunoprecipitation followed by proteomic analysis, but a direct comparison of the interactomes of all three isoforms, and moreover, employing techniques that capture weak or transient interactions, have not been performed. As such, the high degree of similarity amongst the RAS isoforms provides a rather unique opportunity to contrast their presumably very similar interactomes to screen for isoform-specific vulnerabilities. This is especially true for KRAS, given its prominence in human cancers. Using this strategy we show here that the phosphatidylinositol 4-phosphate 5-kinase type-1α (PIP5K1A) is a KRAS-specific interactor that mediates oncogenic KRAS signaling and proliferation.

## Results

### BioID identifies the interactomes of oncogenic RAS isoforms

**Fig. 1:**
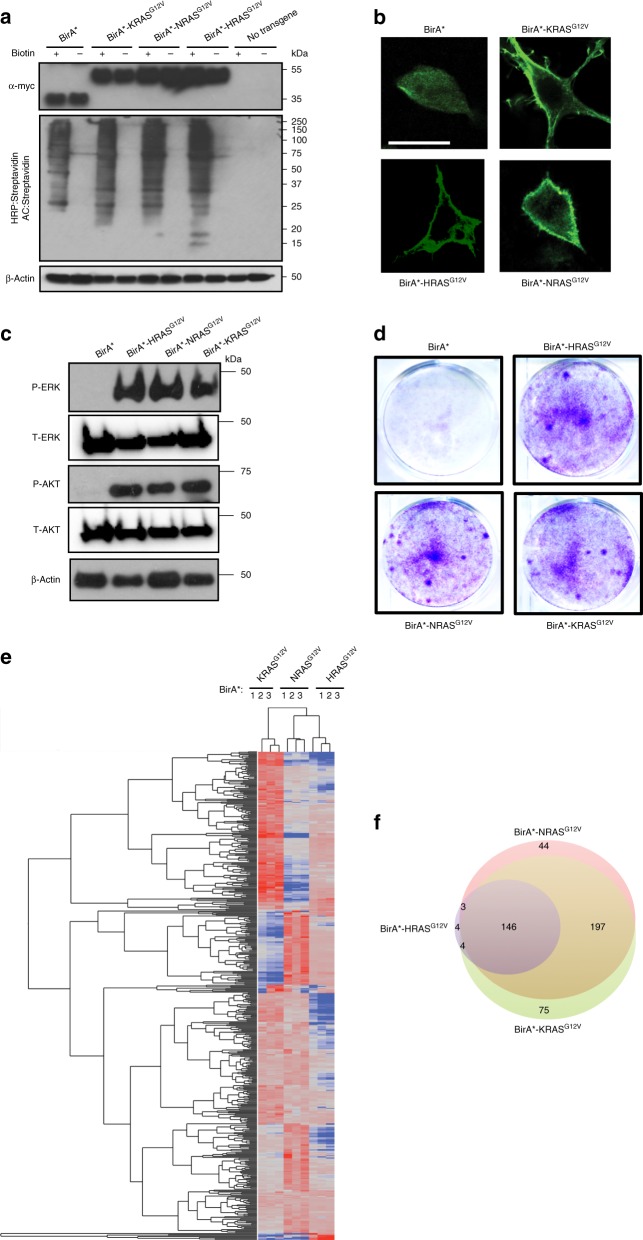
BioID identifies RAS interactomes. **a** Immunoblot detection of myc-tagged BirA* alone or fused to the indicated RAS^G12V^ isoforms (top) and biotinylated proteins with HRP-streptavidin after affinity capture (AC) with streptavidin-conjugated beads (middle) in the absence (−) and presence (+) of biotin. Empty vector-transduced cells (bottom) serve as a negative control. *n* = 2. **b** Representative images indicated myc-tagged BirA* alone or fused to the indicated RAS^G12V^ isoforms, as visualized by fluorescence microscopy. Scale bar: 5 μM. *n* = 1. **c** Immunoblot detection of total (T-) and phosphorylated (P-) ERK and AKT and **d** Crystal Violet staining of cells stably expressing myc-tagged BirA* alone or fused to the indicated RAS^G12V^ isoforms. *n* = 2. **e** Hierarchical clustering of the *z*-scores derived from degree that the 477 interactome proteins were labeled by the indicated myc-tagged BirA*-RAS^G12V^ proteins in triplicate cultures. **f** Venn diagram of the number of interactome proteins identified by BioID by the indicated BirA*-RAS^G12V^ proteins. Where indicated, β-actin serves as a loading control

To assess whether differences in RAS isoforms are manifested in the composition of associating proteins, the interactomes of the oncogenic (G12V) KRAS(4B), NRAS, and HRAS were determined by BirA proximity-dependent biotin identification (BioID)^27^. This approach has the distinct advantage over affinity purification in that it captures weak or transient interactions in living cells^27^. A myc epitope-tagged mutant (R118G) version of the bacterial BirA biotin ligase (termed BirA*), which conjugates biotin to proteins within the immediate vicinity (~10 nm)^28^, was fused in frame to the N-terminus of KRAS^G12V^, NRAS^G12V^, and HRAS^G12V^. To determine if BirA* retained function when fused to RAS proteins, human HEK-HT cells were transiently transduced with an expression vector encoding BirA* as a control or one of the three BirA*-RAS^G12V^ transgenes. These cells critically depend upon RAS oncogenes for transformation and tumorigenesis^5,23,29–31,32,33^, thus any changes observed could be linked to the oncogenic effects of RAS proteins. Immunoblot analysis confirmed expression of each BirA*-RAS^G12V^ protein (Fig. 1a, top). Due to high expression by transient transduction compared to endogenous protein (Supplementary Fig. 1a), it was difficult to distinguish differences in protein levels between the different BirA*-RAS^G12V^ isoforms. However, stable transduction in HEK-HT cells revealed that, as expected^23^, BirA*-HRAS^G12V^ was the highest expressed, followed by BirA*-NRAS^G12V^, with BirA*-KRAS^G12V^ having the lowest expression (Supplementary Fig. 1b). The resultant cells were then treated with or without biotin, after which biotinylated proteins were affinity-captured with streptavidin-conjugated beads, resolved, and immunoblotted with horse radish peroxidase (HRP)-streptavidin. This analysis revealed that all three BirA*-RAS^G12V^-labeled proteins in the presence of biotin (Fig. 1a, middle).

Other proteins have been fused to the N-terminus of RAS proteins, such as GFP^34,35^ and ER^5,36^, without overtly altering their subcellular localization or function. To assess the subcellular localization of the BirA*-RAS^G12V^ proteins, human 293T cells, for ease of visualizing ectopic proteins, were transiently transduced with the four vectors as above and subjected to fluorescence microscopy with an α-myc antibody. This analysis revealed that all three BirA*-RAS^G12V^ proteins were appropriately localized to the plasma membrane (Fig. 1b), the reported primary residency of RAS proteins^24^, and that the cells were similarly transduced (Supplementary Fig. 1c). To assess RAS function in these fusion proteins, lysates from the aforementioned HEK-HT cells stably expressing BirA* or one of the three BirA*-RAS^G12V^ transgenes (Supplementary Fig. 1b) were immunoblotted to detect phosphorylated (T202/Y204) ERK (P-ERK) and phosphorylated (S308) AKT (P-AKT), indicative of activated RAS signaling. This analysis revealed that all three BirA*-RAS^G12V^ proteins increased the level of P-ERK and P-AKT compared to control cells (Fig. 1c). Finally, to assess the oncogenic activity of these fusion proteins, the same cells were seeded in low serum media and colony formation was captured by Crystal Violet staining 5 days later. This analysis revealed that all three BirA*-RAS^G12V^ proteins promoted extensive colony formation (Fig. 1d). Thus, the three BirA*-RAS^G12V^ fusions biotinylated proteins and retained the tested oncogenic RAS functions.

To identify the protein interactomes of the three RAS isoforms, HEK-HT cells were transiently transduced in triplicate with each of the aforementioned vectors encoding BirA* as a control, BirA*-KRAS^G12V^, BirA*-NRAS^G12V^, or BirA*-HRAS^G12V^. High transgene expression characteristic of transient transduction coupled with proximity labeling^27^ was chosen to increase the chance of capturing very weak or transient interactions, although admittedly at the expense of non-specific protein labeling. These 12 derived cultures were treated with biotin, after which the biotin proximity-labeled proteins were affinity captured with streptavidin-conjugated beads and subjected to quantitative bioanalysis by liquid chromatography tandem mass spectrometry (Supplementary Data 1a), similar to approaches previously described for analyzing other proteins^27^. This approach captured 65 proteins reported to associate with RAS from the BioGRID database (Supplementary Data 1b). Among these were the well-described RAS effectors ARAF, BRAF, and RAF1 (CRAF), which directly bind to activated RAS through a Ras-binding domain^37^. The peptide count of recovered proteins was normalized to the level captured by BirA* alone (Supplementary Data 1a). The least recovered RAF isoform (RAF1) was then used as the stringency by which to include biotin-labeled proteins within the RAS interactome, which amounted to at least a 2-fold, significant (*p* < 0.05, Student’s *t* test) enrichment over BirA* cells. Proteins were only considered if at least two separate peptides were identified. By these criteria, a total of 477 endogenous proteins were considered to be specifically labeled (Supplementary Data 2), and as visualized by hierarchical clustering of the derived *z*-score values, in a reproducible fashion (Fig. 1e).

Consistent with the high degree of sequence and functional redundancy amongst the RAS isoforms, three-quarters of identified proteins were labeled by two or all three BirA*-RAS^G12V^ isoforms (Fig. 1f). Network analysis of the 477 labeled proteins revealed two major functional clusters, protein trafficking and signal transduction (Supplementary Fig. 2a). Gene ontology (GO) enrichment analysis similarly identified localization, and sub-categories related to protein and vesicular trafficking and localization, as being the most enriched biological process (Supplementary Data 3). Only a small fraction of proteins were preferentially labeled by a single BirA*-RAS^G12V^ isoform (Fig. 1f and Supplementary Fig. 2b). In summary, BioID revealed that similar proteins were typically labeled by the different BirA*-RAS^G12V^ isoforms, consistent with the above-described high degree of sequence and functional similarities of these proteins, but isoform-specific differences nevertheless exist.

### Interactome components contributing to RAS oncogenesis

The use of ectopic BirA*-RAS^G12V^ to capture weak or transient interactions has the drawback of potentially labeling proteins beyond those associated with RAS isoforms. To identify those captured proteins mediating RAS function, a single-guide RNA (sgRNA) lentiviral library comprised of five different sgRNAs against nearly all (474) of the genes encoding interactome proteins was created. As controls, sgRNAs against 50 essential ribosomal genes and non-targeting sequences were included^38,39^. HEK-HT cells transformed by HRAS^G12V^, NRAS^G12V^, KRAS^G12V^, or as a control no transgene, were stably infected at a low multiplicity with this library. Once the cells were stably infected, genomic DNA was collected for analysis. The cells were then cultured for 2 weeks in low serum to maximize the selection for genes specifically mediating oncogenic RAS function (e.g., compare BirA* to BirA*-Ras^G12V^ cells in Fig. 1d), after which genomic DNA was again collected. The genomic DNA from both time points was PCR amplified, barcoded, and subjected to next-generation sequencing. sgRNA abundance was measured as log 2 ratio of RAS^G12V^-transformed cells to the vector control cells. A score was assigned to all the sgRNAs that were differentially enriched, and the top three enriched sgRNA were averaged for each gene (Supplementary Data 4). sgRNAs targeting the RAS effector BRAF were negatively enriched, consistent with the role of BRAF in promoting oncogenic RAS-driven tumorigenesis^40^, as was *PLK1*, a gene previously reported to be synthetic lethal with oncogenic RAS^41^ (see below).

The targeted genes were roughly evenly split between being positively (31%) and negatively (34%) enriched. As the remainder were unaltered (31%), with a very small number (4%) being both positively or negatively enriched (Supplementary Fig. 3a), the loss-of-function screen reduced the RAS interactome by roughly a third. This analysis also suggests that a large fraction of the proteins identified by BirA* proximity-labeling affect RAS-dependent proliferation (or growth arrest, cell death, differentiation, etc.). Thirty-one percent of the targeted genes were enriched in cells transformed by just one RAS^G12V^ isoform, 21% were enriched in cell transformed by two, 17% were enriched in cell transformed by all three, with the remaining being unaltered (Supplementary Fig. 3b). On the whole, there was no obvious relationship between the degree of proximity labeling and sgRNA enrichment. In summary, BioID identified proteins that positively or negatively affect oncogenic RAS-induced proliferation in both a common and an isoform-specific fashion.

**Fig. 2:**
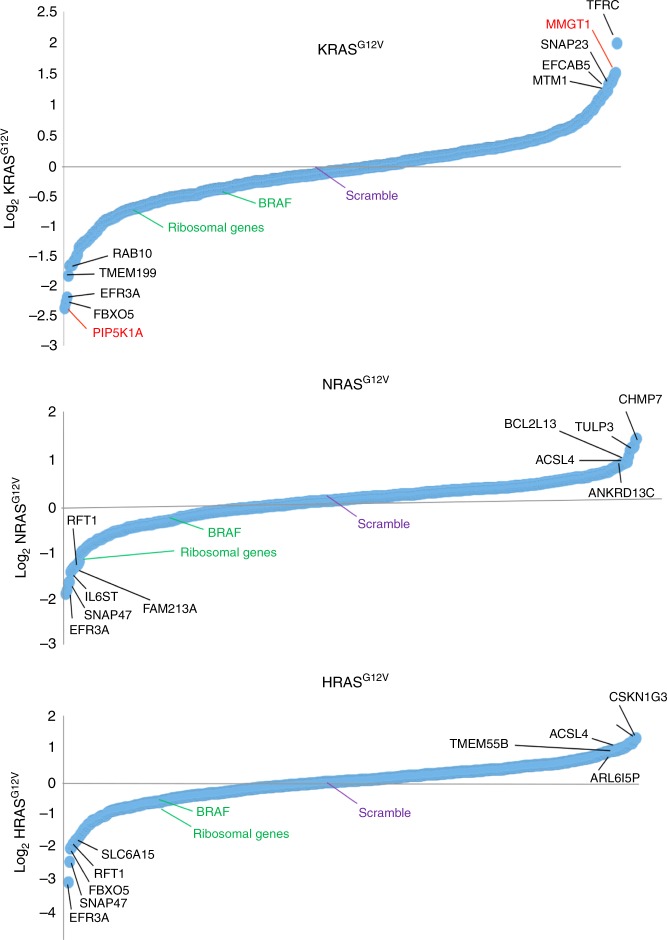
Loss-of-function analysis identifies critical components of the RAS interactomes. Waterfall plot of the sgRNA log_2_ enrichment score of each of the 474 genes encoding interactome proteins targeted by Cas9/CRISPR-mediated loss-of-function (filled circle) in cells transformed by KRAS^G12V^ (top), NRAS^G12V^ (middle), or HRAS^G12V^ (bottom). Enrichment scores for control ribosomal and *BRAF* genes, as well as scramble sgRNA, are shown for reference. Top 1% of positively and negatively enriched genes are labeled (black: enriched in cells transformed by multiple RAS isoforms; red: enriched in cells transformed by only one RAS isoform)

Plotting the enrichment scores for the targeted genes revealed no commonality amongst the top 1% of positively enriched genes. *EFR3A*, *SNAP47*, and *FBXO5* were commonly in the top 1% of negatively enriched genes in cells transformed by NRAS^G12V^, HRAS^G12V^, and/or KRAS^G12V^ (Fig. 2). There was, again, no obvious pattern to these genes, although *EFR3A* is of interest given that it and *PIP5K1A*, the most negatively enriched gene in KRAS^G12V^-transformed cells (Fig. 2), are both involved in phosphatidylinositol signaling^42,43^, a point expanded upon below. Thus, targeting the identified RAS interactomes by CRISPR-Cas9-mediated gene inactivation revealed a host of new proteins mediating oncogenic RAS-driven proliferation in both a common and isoform-specific fashion.

### PIP5K1A specifically associates with oncogenic KRAS

**Fig. 3:**
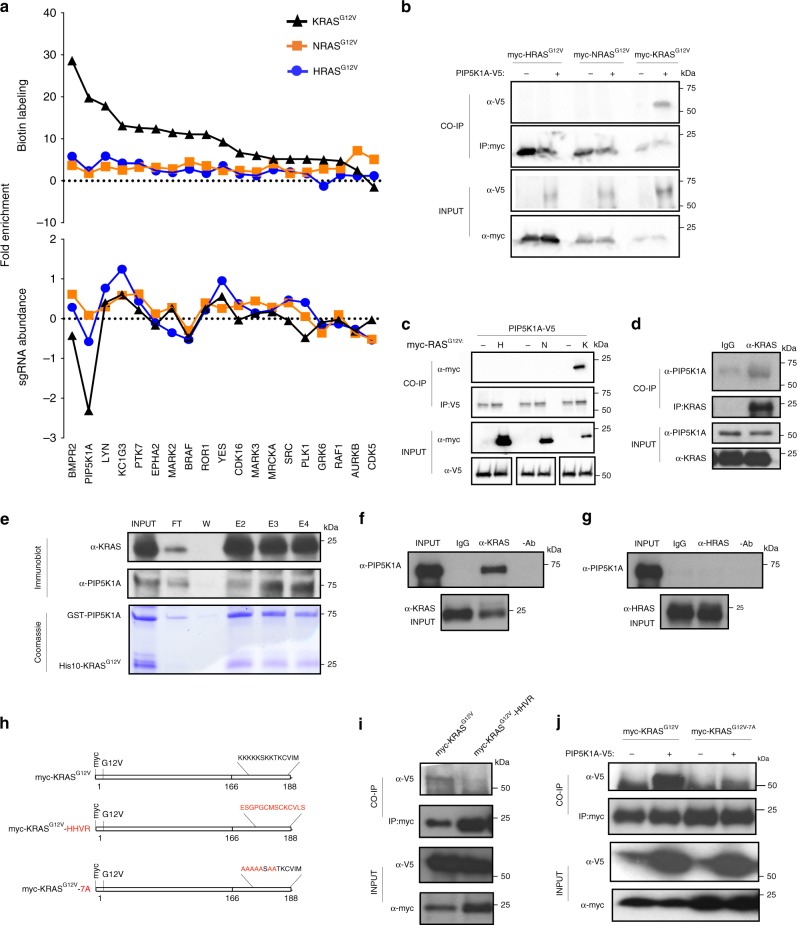
PIP5K1A binds specifically to KRAS^G12V^. **a** Plots of proximity labeling by myc-tagged BirA* fused to (top), and sgRNA enrichment scores in cells transformed with (bottom), KRAS^G12V^ (black triangle), NRAS^G12V^ (orange square), or BirA*-HRAS^G12V^ (blue circle) for the indicated 19 kinases. **b**, **c** Immunoblot detection of the indicated immunoprecipitated (IP) and co-immunoprecipitated (CO-IP) myc-RAS^G12V^ and PIP5K1A-V5 proteins. *n* = 2. **d** Immunoblot detection of IP endogenous KRAS with CO-IP endogenous PIP5K1A from SW620 cells. *n* = 1. **e** Recombinant GST-PIP5K1A and His10-KRAS^G12V^ co-elution (eluates 2–4: E2, E3, and E4) from a Ni-charged column, as detected by Coomassie staining (bottom) or immunoblot (top). INPUT, flow through (FT), and the final wash (W) confirm protein mixtures and retention of proteins on the column. *n* = 2. **f**, **g** Immunoblot detection of IP recombinant **f** His10-KRAS^G12V^ or **g** His10-HRAS^G12V^ with CO-IP recombinant GST-PIP5K1A. *n* = 2. **h** Schematic representation of myc-KRAS^G12V^ unaltered, when the HVR is replaced with that of HRAS (HHVR), and when the indicated seven lysine residues in the HVR are mutated to alanine (7A). **i**, **j** Immunoblot detection of IP myc-KRAS^G12V^ and either **i** myc-KRAS^G12V^-HHVR (*n* = 2) or **j** myc-KRAS^G12V^-7A (*n* = 1) CO-IP with PIP5K1A-V5. Where indicated, INPUT lysates serve to confirm appropriate expression of the indicated proteins, the absence (−) of PIP5K1A-V5 or RAS isoforms serve as a control for the specificity of the CO-IP (note that the α-V5 INPUT immunoblot in **c** was run on a separate gel processed at the same time), and IgG or no antibody serve as a negative control

Depending on the RAS isoform used to transform the cells, there were between 10 to 74 genes more negatively enriched than the control ribosome genes, and even more if benchmarking against the RAS effector *BRAF* (Fig. 2). Given the still large number of potential candidates and the need for druggable targets in RAS signaling, one last enrichment step was performed. Specifically, the degree of BirA*-RAS^G12V^ labeling was compared with the enrichment scores for kinases identified in the RAS^G12V^ interactomes. Nineteen kinases were labeled by one or more BirA*-RAS^G12V^ protein, although typically they were more prevalent in the KRAS^G12V^ interactome (Fig. 3a, top). Benchmarked to the enrichment score of *BRAF*, sgRNAs against five of these kinases were negatively enriched (Fig. 3a, bottom), including the aforementioned RAS synthetic lethal partner PLK1. Of these, the phosphatidylinositol phosphate kinase PIP5K1A was the most attractive candidate, being the second most labeled kinase by BirA*-KRAS^G12V^ (Fig. 3a, top) and the corresponding sgRNAs were the most negatively enriched (Fig. 3a, bottom). In fact, *PIP5K1A* sgRNAs were the most negatively enriched of all the genes targeted, common or unique, in KRAS^G12V^-transformed cells (Fig. 2).

To independently validate the preferential interaction between PIP5K1A and KRAS^G12V^ using a different assay, human 293T cells, chosen for ease of expressing ectopic proteins, were engineered to stably express V5 epitope-tagged PIP5K1A or no transgene and transduced with expression vectors encoding myc epitope-tagged KRAS^G12V^, NRAS^G12V^, or HRAS^G12V^. The myc-RAS^G12V^ oncoproteins were immunoprecipitated with an α-myc antibody, resolved, and PIP5K1A-V5 detected by immunoblot with an α-V5 antibody. This analysis revealed that PIP5K1A-V5 was effectively co-immunoprecipitated (CO-IP) with myc-KRAS^G12V^, but not with myc-NRAS^G12V^ or myc-HRAS^G12V^ (Fig. 3b). To assess the reproducibility of these findings, the experiment was repeated in the reverse direction, demonstrating that myc-KRAS^G12V^, but not myc-NRAS^G12V^ or myc-HRAS^G12V^, CO-IP with PIP5K1A-V5 (Fig. 3c). To assess whether this interaction occurred at the endogenous level, endogenous KRAS was immunoprecipitated from SW620 human *KRAS*-mutant cancer cells, chosen for their high endogenous KRAS expression, with an α-KRAS antibody, followed by immunoblot with an α-PIP5K1A antibody, which confirmed that PIP5K1A CO-IP with KRAS (Fig. 3d). This interaction was validated at the endogenous level in three other *KRAS*-mutant human cancer cell lines (Supplementary Fig. 4). In summary, the preferential labeling of PIP5K1A by BirA*-KRAS^G12V^ compared to the other two RAS isoforms was independently validated by immunoprecipitation assays, and captured at the endogenous level. These findings support PIP5K1A being a unique component of the KRAS^G12V^ interactome.

To determine whether this interaction was direct or indirect, the affinity of recombinant PIP5K1A to recombinant KRAS^G12V^ was assessed. Specifically, recombinant GST-tagged PIP5K1A and His10-tagged KRAS^G12V^ proteins were incubated, complexes captured by affinity to a Ni-charged resin, followed by washes after which eluted proteins were resolved and stained with Coomassie blue as well as immunoblotted with α-PIP5K1A or α-KRAS antibodies. This analysis revealed that both proteins were retained in the same fractions, as assessed by these two detection assays (Fig. 3e). This direct interaction was validated in the opposite direction using a different technique. A mixture of recombinant His10-KRAS^G12V^ and GST-PIP5K1A proteins were subjected to immunoprecipitation with an α-KRAS antibody, followed by immunoblot with α-PIP5K1A or α-KRAS antibodies, which revealed GST-PIP5K1A CO-IP with His10-KRAS^G12V^ (Fig. 3f). To determine if this interaction was isoform specific, the experiment was repeated with His10-HRAS^G12V^, which did not co-immunoprecipitate GST-PIP5K1A (Fig. 3g). To map the site of this isoform-specific interaction in cells, human 293T cells stably expressing PIP5K1A-V5 or no transgene were transduced with a vector encoding either myc-KRAS^G12V^ or myc-KRAS^G12V^-HHVR in which the HVR of KRAS was swapped with that of HRAS (Fig. 3h). Analysis of lysates from these cells revealed that PIP5K1A-V5 CO-IP with myc-KRAS^G12V^, but not the HHVR version (Fig. 3i). PIP5K1A had previously been reported to co-immunoprecipitate with another small GTPase, Rac1. This interaction was dependent upon the C-terminal polybasic region of Rac1^44^, which is similar to the HVR of KRAS(4B)^21^. Taken together, this suggests that the basic residues in the HVR of KRAS may impart some of the binding specificity towards PIP5K1A. To test this, the above co-immunoprecipitation experiment was repeated, except using myc-KRAS^G12V^-7A in which seven lysine residues in the HVR were mutated to alanine (Fig. 3h). This analysis revealed that there was an appreciable reduction in the amount of PIP5K1A-V5 co-immunoprecipitating with myc-KRAS^G12V^-7A (Fig. 3j). In summary, PIP5K1A directly interacts with KRAS in an isoform-specific fashion, including at the endogenous level, through the one region that RAS proteins differ the most, the HVR, due in part to the basic amino acids characteristic of this region in KRAS-4B.

### PIP5K1A mediates oncogenic KRAS signaling and transformation

**Fig. 4:**
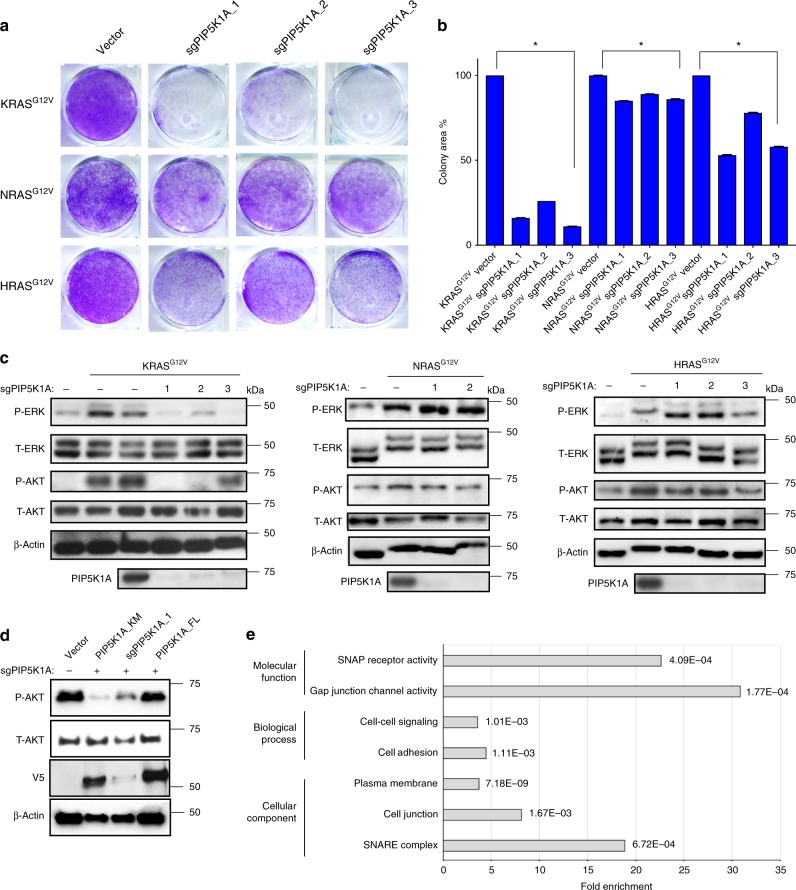
PIP5K1A mediates oncogenic KRAS signaling. **a** Representative Crystal Violet staining, **b** intensity of Crystal Violet staining (mean ± SE, triplicate samples, three experiments, normalized to vector control), and **c** immunoblot detection of phosphorylated (P-) ERK and AKT and PIP5K1A in cells expressing the indicated RAS^G12V^ proteins in the absence (vector) or presence of up to three separate *PIP5K1A* sgRNAs. *p = 0.05, one-sided Student’s *t* test. *n* = 2. **d** Immunoblot detection of P-AKT in Capan-1 cells stably transduced with a vector encoding Cas9 and no sgRNA (−) or *PIP5K1A_1* sgRNA (+) and another vector encoding no transgene (vector), wild-type PIP5K1A, or PIP5K1A^KM^ (detected with an α-V5 antibody). *n* = 2. **e** GO classifications showing fold enrichment and significance (*p* values, Fisher’s exact test) based on GO enrichment analysis of proteins biotinylated by BirA*-KRAS^G12V^ in the absence or in the presence of PIP5K1A. Where indicated, total (T-) ERK and AKT as well as β-actin serve as loading controls

The negative enrichment of *PIP5K1A* sgRNAs specifically in KRAS^G12V^-transformed cells suggests that PIP5K1A is uniquely required for oncogenic KRAS-mediated proliferation. To test this notion, the aforementioned HEK-HT cells stably expressing no transgene or KRAS^G12V^, NRAS^G12V^, or HRAS^G12V^ were stably infected with lentiviral vectors encoding Cas9 and no sgRNA or one of three different sgRNAs targeting the *PIP5K1A* gene. Appropriate expression of PIP5K1A and RAS^G12V^ transgenes was confirmed by immunoblot (see below). The resulting cell lines were seeded at low density and cultured in low serum over the course of 5 days. Crystal Violet staining revealed an 82% average decrease in the number of KRAS^G12V^-transformed cells upon targeting PIP5K1A. In contrast, the average decrease was 13 and 30% in cells transformed by NRAS^G12V^ or HRAS^G12V^, respectively, when PIP5K1A expression was reduced (Fig. 4a, b). To evaluate whether this inhibition of KRAS transformation was a product of reduced oncogenic KRAS signaling, lysates from this panel of cell lines were immunoblotted with antibodies against endogenous PIP5K1A, which confirmed the efficacy of the *PIP5K1A* sgRNAs, as well as P-ERK and P-AKT, to measure oncogenic RAS signaling. This analysis revealed that all three RAS^G12V^ proteins increased P-ERK and P-AKT levels relative to control cells; however, the loss of PIP5K1A only reduced the level of these phosphorylated proteins in KRAS^G12V^-transformed cells (Fig. 4c). In summary, reducing PIP5K1A expression reproducibly suppressed oncogenic KRAS-driven, but not NRAS-driven or HRAS-driven cellular signaling and growth.

### PIP5K1A mediates PI3K and MAPK signaling by oncogenic KRAS

Loss of PIP5K1A reduced P-AKT levels in KRAS^G12V^-transformed cells, suggesting that PIP5K1A affects phosphatidylinositol 3-kinase (PI3K) activity. In agreement, the reduction of P-AKT in *KRAS*-mutant Capan-1 human cancer cells stably induced with *PIP5K1A* sgRNA was reversed upon expressing wild-type PIP5K1A, but not a PIP5K1A^KM^ mutant^45^ in which the presumptive catalytic site was mutated (Fig. 4d). Loss of PIP5K1A also reduced the levels of P-ERK in KRAS^G12V^-transformed cells, suggestive of effects beyond PI3K. We therefore compared the KRAS interactome, determined as above by BirA*-KRAS^G12V^ proximity labeling in HEK-HT cells in triplicate, in the absence and presence of each of three *PIP5K1A* sgRNAs. To filter out general effects of PIP5K1A loss, the same analysis was repeated with BirA*-HRAS^G12V^. Appropriate BirA*-RAS^G12V^ expression and biotin labeling were confirmed by immunoblot analysis (Supplementary Fig. 5a), and GFP-tagged KRAS^G12V^ was shown to have a grossly similar localization in the absence and presence of PIP5K1A (Supplementary Fig. 5b). The loss of PIP5K1A resulted in ~50 proteins enriched (positively or negatively) by proximity labeling with BirA*-KRAS^G12V^. Using the same criteria, half as many proteins were enriched upon the loss of PIP5KIA by proximity labeling with BirA*-HRAS^G12V^, with very little overlap (Supplementary Fig. 5c–f and Supplementary Data 5), suggesting that the KRAS interactome is relatively more sensitive to a loss of PIP5K1A. GO enrichment analysis further revealed that the proteins enriched by BirA*-KRAS^G12V^ proximity labeling in the absence of PIP5K1A were related to membrane localization or dynamics (Fig. 4e).

### PIP5K1A as a potential new target in *KRAS*-mutant cancer

**Fig. 5:**
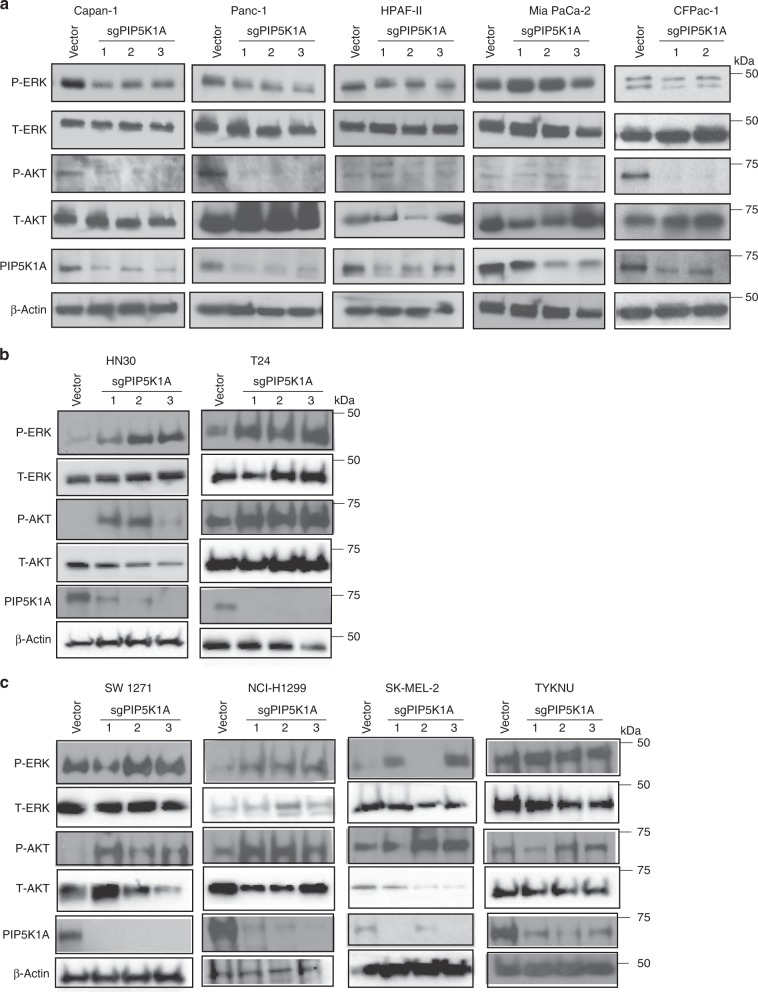
PIP5K1A is an isoform-specific vulnerability of *KRAS*-mutant cancer cells. Immunoblot detection of phosphorylated (P-) ERK and AKT in the indicated **a** *KRAS*-mutant, **b** *HRAS*-mutant, and **c** *NRAS*-mutant human cancer cell lines stably infected with a lentiviral vector encoding Cas9 and no transgene (vector) or one of up to three independent sgRNA targeting *PIP5K1A* (sgPIP5K1A-1 to -3) verified to reduce PIP5K1A expression. *n* = 2. Total (T-) ERK and AKT as well as β-actin serve as loading controls

To address whether the effects observed in KRAS^G12V^-transformed HEK-HT cells are recapitulated in cancer cell lines, PIP5K1A expression was reduced in human pancreatic ductal adenocarcinoma (PDAC) cell lines, given that *KRAS* is mutated in this cancer more than any other^2–4,6^, and the frank lack of therapeutics to treat this disease^46^. The PDAC *KRAS*-mutant cell lines Panc-1, HPAF-II, Capan-1, CFPac-1, and MiaPaca-2 were stably infected with virus derived from the aforementioned lentiviral vector encoding Cas9 and either no transgene or up to three independent *PIP5K1A* sgRNAs. Appropriate reduction of PIP5K1A was confirmed by immunoblot (Fig. 5a). To assess the impact on oncogenic KRAS signaling, the 19 derived cell lines were immunoblotted for P-ERK and P-AKT. This analysis revealed that reducing PIP5K1A with each of the tested *PIP5K1A* sgRNAs resulted in clearly lower P-ERK levels in Capan-1, Panc-1, and HPAF-II cells, a slight decrease in CFPac-1 cells, but no effect in MiaPaCa-2 cells (Fig. 5a). P-AKT levels were similarly lower in Capan-1, Panc-1, and CFPac-1 cells, but appeared unchanged in HPAF-II and MiaPaCa-2 cells, although P-AKT immunoreactivity was extremely low (Fig. 5a). As such, loss of PIP5K1A reduced endogenous oncogenic KRAS signaling, although not in every cell line. To address whether this effect was unique to *KRAS*-mutant human cancer cells, PIP5K1A expression was similarly reduced by Cas9-mediated gene inactivation, as confirmed by immunoblot analysis, with three separate *PIP5K1*A sgRNAs in the *HRAS*-mutant human cancer cell lines HN30 and T24 (Fig. 5b) and in the *NRAS*-mutant human cancer cell lines SW1271, NCI-H1299, SK-MEL-2, and TYKNU (Fig. 5c). Immunoblot analysis of these 18 cell lines and their six vector control counterparts revealed that reducing PIP5K1A generally either increased or had no effect on the levels of P-ERK or P-AKT. The only deviation was one *PIP5K1A* sgRNA out of the three tested reduced P-ERK levels in SK-MEL-2 cells and P-AKT levels in TYKNU cells (Fig. 5b, c). As such, PIP5K1A mediates endogenous oncogenic KRAS signaling in what appears to be an isoform-specific fashion.

**Fig. 6:**
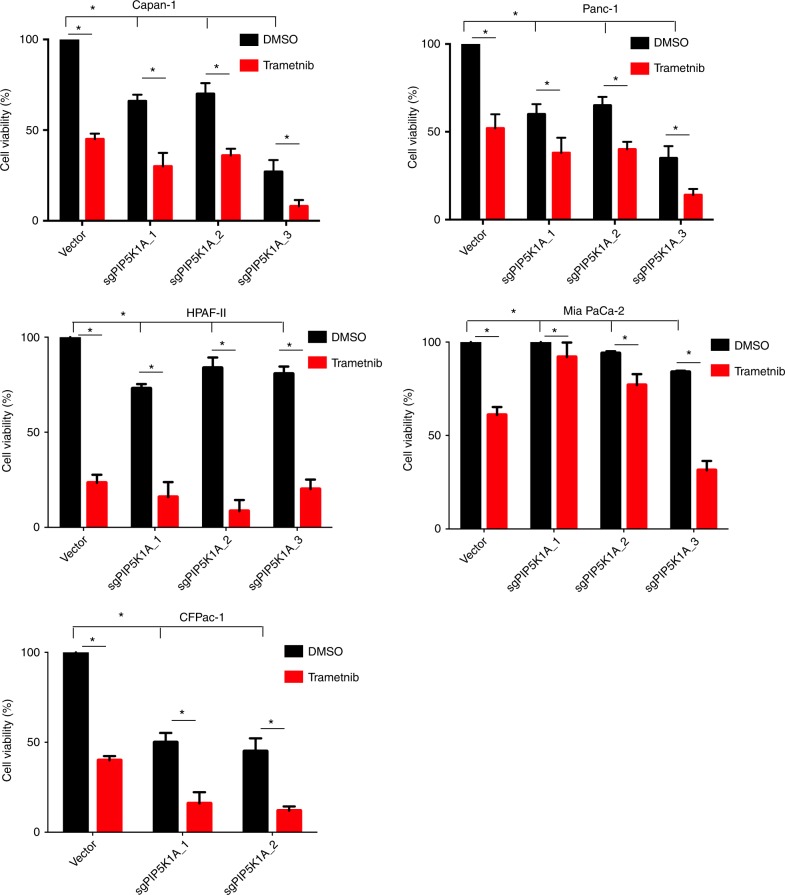
PIP5K1A loss is additive with a MEK inhibitor in *KRAS*-mutant cancer cells. Percent cell viability (mean ± SD, triplicate samples, two experiments, normalized to vector control cells treated with DMSO) of the indicated *KRAS*-mutant human cancer cell lines stably infected with a lentiviral vector encoding Cas9 and no transgene (vector) or one of up to three independent sgRNA targeting *PIP5K1A* (sgPIP5K1A-1 to -3) after treatment with vehicle (DMSO) or 25 nM of trametinib for 72 h. *p = 0.05, two-way ANOVA test

**Fig. 7:**
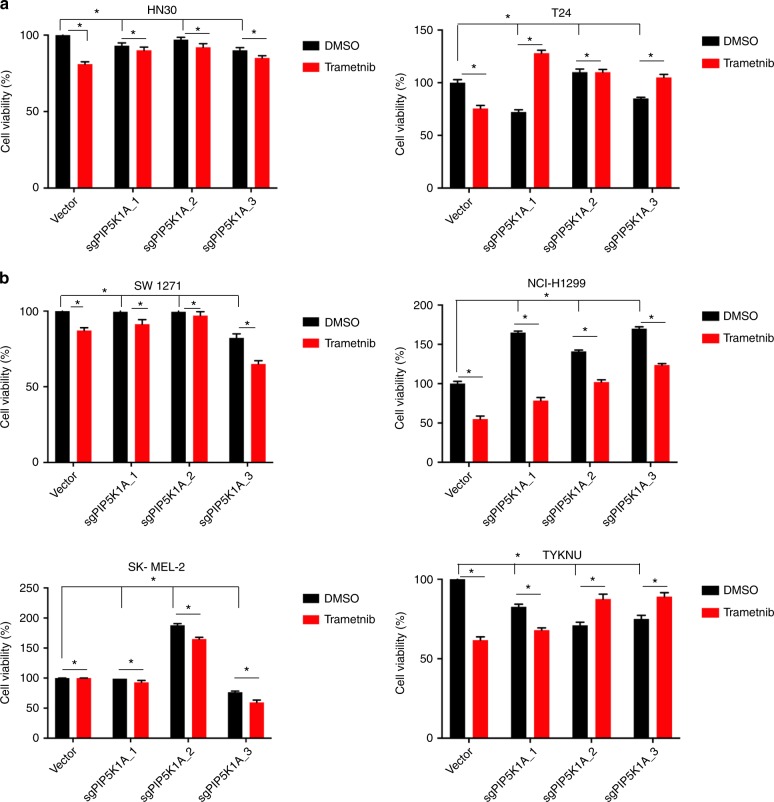
PIP5K1A loss has minimal impact on *HRAS*-mutant or *NRAS*-mutant cancer cells. Percent cell viability (mean ± SD, triplicate samples, two experiments, normalized to vector control cells treated with DMSO) of the indicated **a** *HRAS*-mutant and **b** *NRAS*-mutant human cancer cell lines stably infected with a lentiviral vector encoding Cas9 and no transgene (vector) or one of up to three independent sgRNA targeting *PIP5K1A* (sgPIP5K1A-1 to -3) after treatment with vehicle (DMSO) or 25 nM of trametinib for 72 h. *p = 0.05, two-way ANOVA test

To assess whether reducing PIP5K1A negatively impacts cellular proliferation of specifically *KRAS*-mutant human cancer cell lines, the above 43 derived cell lines were assayed twice in triplicate for changes in the number of metabolically active (viable) cells using the CellTiterGlo luminescence assay. This analysis revealed that in all but MiaPaCa-2 cells, a reduction of PIP5K1A led to a 40% average decrease of viable cells in the *KRAS*-mutant PDAC cell lines (Fig. 6). In contrast, reduction of PIP5K1A in the *HRAS*-mutant and *NRAS*-mutant human cancer cell lines typically either had no consistent effect or even an increase in the number of viable cells, with the exception of TYKNU cells, in which all three *PIP5K1A* sgRNAs led to a decrease in cell number (Fig. 7a, b). With the caveat that the cell lines tested were derived from different cancers, PIP5K1A mediates growth driven by endogenous oncogenic KRAS in what appears to be an isoform-specific fashion.

Finally, the clinical MEK inhibitor trametinib is highly effective at suppressing the mitogen-activated protein kinase (MAPK) arm of oncogenic RAS signaling, but to date has proven ineffective for the treatment of *KRAS*-mutant PDAC^47,48^. To test if reducing PIP5K1A to dampen oncogenic KRAS signaling could enhance the antineoplastic effect of trametinib, the aforementioned panel of *KRAS*-mutant, *HRAS-*mutant, and *NRAS*-mutant human cancer cell lines in which PIP5K1A expression was unaltered or reduced were treated with or without trametinib. Reduction of PIP5K1A combined with trametinib reduced the number of viable cells more than either change alone in three of the five tested *KRAS*-mutant human cancer cell lines (Fig. 6). In contrast, loss of PIP5K1A either had no effect or even increased the number of viable cells in all but one (TYKNU) of the *HRAS*-mutant or *NRAS*-mutant human cancer cell lines, and even in this one cell line, the effect was blunted (Fig. 7a, b). In summary, PIP5K1A loss reduces oncogenic KRAS signaling, which could potentially be capitalized upon to add specificity to MAPK inhibitors for the treatment of *KRAS*-mutant cancers like PDAC.

## Discussion

We leveraged the high amino acid identity between the RAS isoforms to search for isoform-specific vulnerabilities, first by determining the presumptive interactomes of each RAS isoform by BioID, and then second, by inactivating the corresponding genes in cells transformed by each of the three RAS oncogenes. In detail, BioID revealed that most of the labeled proteins were related to protein trafficking or signal transduction, the main occupation of RAS proteins. The labeled proteins were common to multiple RAS interactomes, but there was a small subset that were isoform-specific, supporting the contention that differences in the sequence of RAS proteins can manifest in the interactome. Admittedly, interpreting these interactomes is tempered because some of the proteins labeled may reflect the local milieu rather than RAS function per se, and the interactomes were derived from BirA*-RAS^G12V^ proteins over-expressed in a single cell line. The secondary CRISPR-Cas9 functional screen thus served as a critical filter, weeding out about a third of the interactome, namely proteins that did not affect oncogenic RAS-driven proliferation. There was a fair degree of discordance between the two datasets (proteins identified in the interactome of one RAS isoform by BioID were important for growth of cells transformed by a different RAS isoform in the CRISPR-Cas9 screen, etc.). This may be a product of labeling proteins within the proximity of RAS^G12V^ rather than being bona fide interactome proteins, using ectopically expressed RAS for proximity labeling or to drive proliferation, assaying for just one phenotype (cell proliferation) in the CRISPR-Cas9 screen, combinations of these, or for other reasons. Nevertheless, the approach successfully identified PIP5K1A to preferentially bind to, and mediate signaling and proliferation by KRAS^G12V^. This suggests that PIP5K1A could be used as a benchmark to identify other potential vulnerabilities from this screen. In this regard, we identified 57 proteins enriched at least half as well as PIP5K1A in both assays (Supplementary Data 6).

The top isoform-specific candidate from this analysis, PIP5K1A, joins a very small group of proteins like galectin-3^49^ and calmodulin^50^ and others reported to bind to a specific RAS isoform. Mechanistically, PIP5K1A appeared to directly bind to KRAS though the very region that differs amongst RAS isoforms, the HVR, thereby providing one explanation to this specificity. This binding was mediated, at least in part, through basic amino acids in the C-terminus of the HVR. PIP5K1A has also been found to associate with other small GTPases (Rac1 and ARF6)^44,51^, although Rac1 appears to have the highest affinity^44^.

Loss of PIP5K1A reduced the phosphorylation of AKT induced by ectopic and endogenous oncogenic KRAS, but not NRAS or HRAS. PIP5K1A phosphorylates the fifth position in the inositol ring of PtdIns(4)*P* to produce PtdIns(4,5)*P*_2_ (PIP2)^52^, which is a substrate for class I PI3Ks^53^, known RAS effectors^54^. PI3K phosphorylates the third position of PtdIns(4,5)*P* to produce PtdIns(3,4,5)*P*_3_ (PIP3)^52^. PDK1 (PDPK1) and AKT bind PIP3 at membranes vis-à-vis their PH domains, leading to activating phosphorylation of AKT1 on S308 by PDK1^55^. Although other interpretations are possible, based on this the simplest model is that PIP5K1A promotes oncogenic KRAS signaling by supplying PI3K with its substrate PIP2. Three lines of evidence favor this notion. First, PI3K binds to activated RAS^54^, which may place it within the vicinity of PIP2 generated by KRAS-associated PIP5K1A. Second, PIP5K1A promotes PI3K activity, at least as measured by S308 phosphorylation of AKT. Third, PIP5K1A^KM^ failed to rescue the reduction in P-AKT upon targeting endogenous PIP5K1A, supporting the contention that activation of the PI3K/AKT pathway by oncogenic KRAS depends upon the kinase activity of PIP5K1A. It is also worth pointing out that *EFR3A*, one of the most commonly negatively enriched genes identified in the CRISPR-Cas9 interactome screen, serves as a plasma membrane anchor for PI4PKIIIα, the lipid kinase that produces PtdIns(4)*P*, a substrate of PIP5K1A^42^. Furthermore, oncogenic RAS was recently shown to suppress the anti-proliferative effects resulting from knocking down or pharmacologically inhibiting PIP4K lipid kinases^56^.

Loss of PIP5K1A also reduced the phosphorylation of ERK induced by ectopic and endogenous oncogenic KRAS, but not NRAS or HRAS. It is possible that this is a product of cross-talk between the PI3K/AKT and MAPK pathways, for example, through wild-type RAS proteins^32^, suppressing the inhibitory PI3K1P1 by the MAPK pathway^56^, and so on, although admittedly this is a complex relationship^57^. Alternatively, the reduction of P-ERK may point to an effect on KRAS itself. In this regard, the loss of PIP5K1A altered the proximity labeling by BirA*-KRAS^G12V^ of membrane proteins and proteins involved in membrane dynamics. Perhaps then the loss of PIP5K1A disrupts the localization of KRAS^G12V^ from regions of the plasma membrane undergoing dynamic remodeling, thereby reducing proximity to signaling proteins or spatially constricting KRAS to microdomains that inefficiently transmit signaling. In support, phosphatidylinositols were recently reported to play a more direct role in oncogenic KRAS signaling. Specifically, targeting the lipid phosphatase Pseudojanin to the plasma membrane to reduced PtdIns(4)*P* and PtdIns(4,5)*P*_2_ redistributed GFP when conjugated to the HVR of KRAS, but not to the HVR of HRAS, away from the plasma membrane^58^. Nevertheless, how loss of PIP5K1A reduces MAPK signaling by KRAS remains to be determined.

Reducing the expression of PIP5K1A in both oncogenic KRAS-transformed HEK-HT cells and four of five PDAC cell lines decreased cell numbers while largely having no negative effect on the oncogenic *HRAS* or *NRAS* positive cell lines. Nevertheless, one PDAC cell line was unaffected. Perhaps related, PIP5K1A was also not identified in whole-genome CRISPR-Cas9 loss-of-function screens of *KRAS*-mutant cancer cell lines (e.g., ref.^59^). As such, PIP5K1A may not be a single vulnerability for all *KRAS*-mutant cancers, a point that needs further investigation, especially in vivo. The value of inhibiting PIP5K1A may instead lie in capitalizing on the unique requirement of this kinase for oncogenic KRAS signaling to impart specificity to inhibitors of RAS effector pathways. Indeed, reducing PIP5K1A was additive to the antineoplastic effect of trametinib in four of five PDAC cell lines. The fact that knockout mice of the murine counterpart of human PIP5K1A (*Pip5k1b*) are viable^60^ and that the encoded protein is druggable^61^ makes this possibility even more attractive.

In conclusion, we leveraged the high similarity of the different RAS proteins for BioID analysis coupled to a loss-of-function screen to interrogate the oncogenic activity of the different RAS isoforms. This approach identified PIP5K1A as specifically mediating oncogenic KRAS signaling and growth, supporting the contention that RAS isoforms may function differently through unique protein interactions. Further, as PIP5K1A is druggable, it may provide a way to target oncogenic KRAS, perhaps in conjunction with inhibitors of RAS effector pathways.

## Methods

### Primers

A complete list of primers and their sequences used in this study are provided in Supplementary Data 7.

### Plasmid construction

For BirA* proximity-labeling experiments, human *KRAS*^*G12V*^ (4B splice variant), *NRAS*^*G12V*^, and *HRAS*^*G12V*^ cDNAs were created by gene synthesis and cloned in frame 3′ of myc-BirA* in the plasmid pcDNA3.1mycBioID (Addgene vector 35,700)^27^, creating plasmid pcDNA3.1-myc-BirA*-KRAS^G12V^, pcDNA3.1-myc-BirA*-NRAS^G12V^, and pcDNA3.1-myc-BirA*-HRAS^G12V^. For analysis of cellular signaling by BirA*-RAS proteins, these cDNAs were subcloned into mycBioID-pBabePuro (Addgene vector 80,901)^62^, creating plasmids mycBioID-pBabePuro-myc-BirA*KRAS^G12V^, -NRAS^G12V^, and -HRAS^G12V^. To create cell lines for CRISPR-Cas9 screens, the above *KRAS*^*G12V*^, *NRAS*^*G12V*^, and *HRAS*^*G12V*^ cDNAs were PCR amplified to incorporate an N-terminal myc epitope tag and cloned into pWZL-Blast (a kind gift of Jay Morgenstern), creating plasmids pWZL-Blast-myc-KRAS^G12V^, -NRAS^G12V^, and -HRAS^G12V^. For co-immunoprecipitation experiments from cells, the above *myc-KRAS*^*G12V*^, *myc-NRAS*^*G12V*^, and *myc-HRAS*^*G12V*^ cDNAs were cloned into pBabePuro (Addgene vector 1764)^62^, creating plasmids pBabePuro-myc-KRAS^G12V^, -NRAS^G12V^, and -HRAS^G12V^. pLX304-PIP5K1A-V5 was provided by the Duke RNAi core^63^. For interaction studies with recombinant proteins, the above *KRAS*^*G12V*^ and *HRAS*^*G12V*^ cDNAs were PCR amplified to incorporate an N-terminal, 10× His sequence and cloned into pET21a (EMD Millipore), creating plasmids pET21a-His10-KRAS^G12V^ and -HRAS^G12V^. *PIP5K1A* cDNA from pLX304-PIP5K1A-V5 was PCR amplified to incorporate GST N terminally and cloned into pGEX-6P2 (GE Healthcare), creating plasmid pGEX-6P2-GST-PIP5K1A. For mapping the interactions between KRAS and PIP5K1A, human *KRAS*^*G12V*^ (4B splice variant) in which the sequence encoding the last 23 amino acids was replaced with that of HRAS or the sequence encoding the most terminal seven lysine residues were mutated to encode alanine were created by gene synthesis, PCR amplified to incorporate an N-terminal myc epitope tag, and cloned into pBabePuro, creating plasmids pBabePuro-myc-KRAS^G12V^-HHVR and -7A. To genetically ablate the *PIP5K1A* gene, the sequence encoding three independent sgRNA (5′-AAGGCTCAACCTACAAACGG, 5′-ATCTGGAATCAAGAGACCCA, 5′-AGAACCTCAACCAGAACCCT) targeting *PIP5K1A* were cloned in LentiCRISPR v2.0 (Addgene vector 52,961)^64^, creating plasmids LentiCRISPR2.0-sgPIP5K1A-1, -2, and -3. PIP5K1A kinase mutant (PIP5K1A^KM^) was generated by mutating residues K193R, D322A, and D404A of PIP5K1A. These mutations are analogous to those created in zebrafish PIP5K1A (Supplementary Fig. 6) that inactivated the kinase activity of this enzyme^45^. *PIP5K1A* and *PIP5K1A*^*KM*^ was PCR amplified to include a C-terminal V5 epitope tag and subcloned into pWZL-Blast, creating plasmids pWZL-Blast-PIP5K1A-V5 and PIP5K1A^KM^-V5. To visualize KRAS in the absence of PIP5K1A, the above *KRAS*^*G12V*^ cDNA was cloned into the plasmid pDEST-53 (Invitrogen) by gateway cloning to create plasmid pDEST-53-KRAS^G12V^.

### Cell culture

HEK-HT, human embryonic kidney (HEK) epithelial cells ectopically expressing hTERT and the early region of SV40^29^, were not authenticated or confirmed to be free of mycoplasma infection. The 293T cells were obtained from the Duke University Cell Culture Facility were authenticated by short tandem repeat (STR) profiling using the GenePrint10 system (Promega Corporation) and were confirmed to be free of mycoplasma infection, as assessed by the Duke Cell Culture Facility using MycoAlert PLUS test (Lonza). Both cell lines were cultured in Dulbecco's modified Eagle's medium (DMEM) supplemented with 10% fetal bovine serum (FBS) and 1% penicillin–streptomycin. The *KRAS*-mutant human cancer lines CFPac-1, HPAF-II, Capan-1, MiaPaca-2, Panc-1, SW620, H727, and AsPc-1 cells were provided by the Duke University Cell Culture Facility were authenticated by STR profiling and confirmed to be free of mycoplasma infection as above. CFPac-1 cells were cultured in Iscove’s modified Dulbecco’s medium (IMDM) supplemented with 10% FBS. HPAF-II cells were cultured in minimum Eagle’s medium supplemented with 10% FBS, 1% non-essential amino acid, and 1% sodium pyruvate. Panc-1 cells were grown in DMEM supplemented with 10% FBS. Capan-1 cells were grown in IMDM supplemented with 20% FBS. MiaPaca-2 cells were grown in DMEM supplemented with 10% FBS and 2.5% equine serum. The *NRAS*-mutant human cancer lines SK-MEL-2, TYKNU, NCI-H1299, and SW1271 were kind gifts of by Drs. Kris Wood (Duke University, USA) and Channing Der (University of North Carolina at Chapel Hill, USA) and were authenticated by STR profiling and confirmed to be free of mycoplasma infection by these labs. SK-MEL-2 cells were cultured in DMEM medium supplemented with 10% FBS. TYKNU, NCI-H1299, and SW1271 cells were grown in RPMI medium supplemented with 10% FBS. The *HRAS*-mutant human cancer lines T24 and HN30 were provided by the Duke University Cell Culture Facility and as a kind gift from Dr. Adrienne Cox (University of North Carolina at Chapel Hill, USA), respectively, were authenticated by STR profiling and confirmed to be free of mycoplasma infection as above or by the aforementioned lab. T24 were cultured in McCoy’s 5A medium supplemented with 10% FBS. HN30 cells were cultured in DMEM medium supplemented with 10% FBS.

### Immunoblot

To detect BirA*-RAS^G12V^ proteins and biotin labeling, HEK-HT cells or HEK-HT cells stably infected with LentiCRISPR2.0 or LentiCRISPR2.0-sgPIP5K1A-1, -2, or -3 were transiently transfected with pcDNA3.1-myc-BirA*-KRAS^G12V^, -NRAS^G12V^, and/or -HRAS^G12V^ were treated with biotin (see the Biotin identification section below) and then lysed in buffer containing 1% NP-40, 100 mM Tris-HCl, pH 8.0, 50 mM NaCl, 1 mM EDTA, 1 mM phenylmethylsulfonyl fluoride (PMSF), and protease inhibitors (Roche) by end-to-end incubation at 4 °C for 30 min. Lysates were centrifuged at 21,000 x *g* for 10 min. Total protein was quantified using BCA kit (Thermo Fisher Scientific). Proteins were separated on 12.5% or gradient sodium dodecyl sulfate-polyacrylamide gel electrophoresis (SDS-PAGE) gels (Bio-Rad) and transferred onto polyvinylidene difluoride membranes and probed with anti-myc (Cell Signaling, #2276; 1:1000) or β-actin (Cell Signaling #3700; 1:5000) primary antibodies in blocking buffer containing either 5% milk or 5% bovine serum albumin followed by the secondary antibodies of goat anti-rabbit IgG (H+L) HRP (Thermo Fisher Scientific, #65-6120, 1:2000) or goat anti-mouse IgG (H+L) HRP (Life Technologies, #G21040, 1:5000). Immunoblots were visualized using ECL Western Blotting Detection Reagent (Amersham) followed by either exposure to either X-ray film and converted to a gray-scale image with an Epson V370 Photoscanner or digital acquisition using Chemi Doc Imager (Bio-Rad). Images were cropped and in some cases the contrast and/or brightness were altered equally across the entire cropped image for displaying as figures. The unaltered images are provided as Supplementary Figures for comparison. The same lysates were processed as described above to affinity capture biotinylated proteins, resolved, and immunoblotted with HRP-Streptavidin (Thermo Fisher Scientific, #22130; 1:10,000).

To detect phosphorylated (P-) and total (T-) ERK and AKT in HEK-HT cells stably expressing BirA* or BirA*-RAS^G12V^ proteins, HEK-HT cells were stably infected with retroviruses^65^ derived from mycBioID-pBabePuro or mycBioID-pBabePuro-myc-BirA*KRAS^G12V^, -NRAS^G12V^, and -HRAS^G12V^. Specifically, a mixture of 3 μg of the plasmids pCL-10A1 and one of the above mycBioID-pBabePuro or mycBioID-pBabePuro-myc-BirA*KRAS^G12V^, -NRAS^G12V^, and -HRAS^G12V^ with 18 μl Fugene was added to the media of 293T cells plated in a 10 cm dish. After 48 h, 10 ml of retrovirus-containing media were filtered (0.45 μm filter), mixed with 10 μl of 10 mg/ml polybrene (Sigma), and used to refresh the media of HEK-HT plated in a 10 cm plate. The process was repeated 24 h later, and then another 24 h later, the media of the infected HEK-HT cells were refreshed with media containing 1 μg/ml puromycin. Cells were selected for puromycin resistance over the course of 7 days. Stably transduced cells were cultured in low serum (0.5%) for 24 h to evaluate RAS signaling. Lysates were the prepared, resolved, immunoblotted, and visualized as above with anti-P(308)-Akt (Cell Signaling, #9275; 1:500), P-ERK (Cell Signaling, #4370; #9101; 1:1000), Akt (Cell Signaling, #9272; 1:1000), ERK (Cell Signaling, #9102; 1:1000), and β-actin primary antibodies. In some cases, membranes were cut, stripped (which was confirmed by exposure to film), and reprobed to detect total ERK, AKT, and actin. To detect phosphorylated (P-) and total (T-) ERK and AKT as well as PIP5K1A in HEK-HT cells transformed by KRAS^G12V^, -NRAS^G12V^, or -HRAS^G12V^ in the absence and presence of PIP5K1A, the above HEK-HT cells stably transduced with LentiCRISPR2.0 or LentiCRISPR2.0-sgPIP5K1A-1, -2, or -3 were infected with retrovirus derived from pWZL-Blast-myc-KRAS^G12V^, -NRAS^G12V^, or -HRAS^G12V^ and selected in 1 μg/ml blasticidin for 5 days as above. Lysates were prepared, resolved, and immunoblotted as above with P-Akt, P-ERK, Akt, ERK, PIP5K1A (Cell Signaling, #9396; 1:500), and β-actin primary antibodies, as above. To detect phosphorylated (P-) and total (T-) AKT as well as ectopic PIP5K1A wild type of KM mutant in cells lacking PIP5K1A, Capan-1 cells were stably infected with lentiviruses derived from LentiCRISPR2.0 (vector) or LentiCRISPR2.0-sgPIP5K1A-1, and then retroviruses derived from pWZL-Blast (vector), pWZL-Blast-PIP5K1A-V5, or PIP5K1A^KM^-V5, as above. Lysates were prepared, resolved, and immunoblotted with P-Akt, Akt, V5, and β-actin primary antibodies as above. To detect changes in PIP5K1A, P-ERK, and P-AKT in human cancer cell lines in the absence and presence of PIP5K1A, Capan-1, Panc-1, HPAF-II, MiaPaCa-2, CFPac-1, HN30, T24, SW1271, NCI-H1299, and SK-MEL-2, TYKNU were stably infected with lentiviruses derived from LentiCRISPR2.0 or LentiCRISPR2.0-sgPIP5K1A-1, -2, or -3 and immunoblotted for P-AKT, Akt, P-ERK, ERK, PIP5K1A, and β-actin as above. Uncropped immunoblots are shown in Supplementary Fig. 7.

### Immunofluorescence and fluorescence microscopy

To visualize BirA*-RAS proteins in cells, 293T cells were plated on glass coverslips and transiently transfected with pcDNA3.1mycBioID or pcDNA3.1-myc-BirA*-KRAS^G12V^, -NRAS^G12V^, or -HRAS^G12V^ using the Fugene 6 reagent according to the manufacturer’s instructions (Promega Corporation). Forty-eight hours later, cells were fixed, permeabilized, and incubated with primary α-myc antibody (Cell Signaling, #2276) overnight at 4 °C. Cells were then washed five times in 1× phosphate-buffered saline (PBS) and incubated with Alexa Fluor 488-conjugated secondary antibody for 2 h, after which cells were washed five times in 1× PBS. The cells were visualized at ×20 or ×100 magnification using confocal microscopy with a Leica DMI6000CS scanning confocal imaging system equipped with Leica Plan Apochromat that captured images at a resolution of 600 dpi. To visualize KRAS in *PIP5K1A* sgRNA-treated cells, 293T cells were stably infected with LentiCRISPR2.0 (vector) or LentiCRISPR2.0-sgPIP5K1A-1 as above, then transiently transfected with pDEST-53-KRAS^G12V^ (encoding GFP-KRAS^G12V^). Forty-eight hours later, cells were fixed and GFP-KRAS^G12^ visualized at ×20 magnification using confocal microscopy as above.

### Cell proliferation

A total of 1.5 × 10^4^ HEK-HT cells stably transduced with mycBioID-pBabePuro or mycBioID-pBabePuro-myc-BirA*KRAS^G12V^, -NRAS^G12V^, and -HRAS^G12V^ or stably transduced with pWZL-Blast-myc-KRAS^G12V^, -NRAS^G12V^, or -HRAS^G12V^ and one of LentiCRISPR2.0 (vector) or LentiCRISPR2.0-sgPIP5K1A-1, −2, or -3 (see Immunoblot section above) were seeded in triplicate into 6-well plates cultured in low (0.5% FBS) serum. After 5 days, cells were stained with Crystal Violet (Sigma-Aldrich) in 0.04% Crystal Violet in 20% ethanol to visualize cells. A total of 2 × 10^3^ Capan-1, Panc-1, HPAF-II, MiaPaca-2, CFPac-1, HN30, T24, SW1271, NCI-H1299, SK-MEL-2, and TYKNU cells stably transduced with LentiCRISPR2.0 (vector) or LentiCRISPR2.0-sgPIP5K1A-1, -2, or -3 were seeded in triplicate into 96-well plates, two independent times. Twenty-four hours later, the cells were supplemented with fresh medium containing 25 nM trametinib (Chemietek) or dimethyl sulfoxide (DMSO) as a vehicle control. Seventy-two hours later, cell viability was measured using Cell Titer Glo (Promega Corporation) reagent according to the manufacturer’s protocol. Percentage cell viability was calculated as 100% × (absorbance of the treated wells − absorbance of the blank control wells)/(absorbance of the negative control wells − absorbance of the blank control wells).

### Biotin identification

For the first proximity-labeling experiment, HEK-HT cells were transiently transfected in triplicate with pcDNA3.1mycBioID, pcDNA3.1-myc-BirA*-KRAS^G12V^, -NRAS^G12V^, and -HRAS^G12V^ using the Fugene 6 reagent according to the manufacturer's instructions (Promega Corporation). For the second proximity-labeling experiment polyclonal populations of HEK-HT cells stably infected with lentivirus derived from LentiCRISPR v2.0 (vector) or LentiCRISPR2.0-sgPIP5K1A-1, -2, or -3 (see below) were transiently transfected in triplicates (three replicates each of three separate *PIP5K1A* knockout lines) with pcDNA3.1-myc-BirA*-KRAS^G12V^ or pcDNA3.1-myc-BirA*-HRAS^G12V^ as above. Twenty-four hours after transfection, 50 μM biotin was added to the cultures, and then 24 h later, the cells were harvested, lysed, and biotinylated proteins captured by streptavidin-conjugated beads^27^. Briefly, cells were lysed at 4 °C with end-to-end rotation for 30 min in lysis buffer containing 50 mM Tris-HCl (pH 7.5), 500 mM NaCl, 0.2% SDS, 1 mM dithiothreitol, 1 mM PMSF, and protease inhibitor tablet (Roche). The cell lysate was incubated with magnetic streptavidin beads at 4 °C overnight. Next day, the streptavidin beads were washed at room temperature for 8 min on a rotator first with Wash Buffer 1 (2% SDS (w/v)), then with Wash Buffer 2 (0.1% (w/v) deoxycholic acid, 1% (w/v) Triton X-100, 1 mM EDTA, 500 mM NaCl, 50 mM 4-(2-hydroxyethyl)-1-piperazineethanesulfonic acid (HEPES), pH 7.5), and finally with Wash Buffer 3 (0.5% (w/v) deoxycholic acid, 0.5% NP-40, 1 mM EDTA, 250 mM LiCl). Biotinylated proteins were liberated from the streptavidin-conjugated beads by heating the 95 °C for 5 min in SDS-PAGE buffer containing 2 mM biotin.

Fifty microliters of samples were separated by SDS-PAGE (4–12% Invitrogen NuPAGE; MES buffer) for 6 min followed by fixation and Coomassie staining. Bands were excised and peptides isolated by in-gel tryptic digestion. After taking peptide extracts to dryness in Maximum Recovery LC vials (Waters), samples were reconstituted in 40 μl of 1% trifluoroacetic acid/2% acetonitrile containing 25 fmol/μl yeast alcohol dehydrogenase surrogate standards. A QC pool was prepared by mixing equal volumes of all samples.

Four microliters of peptide digests (~10% of each sample) were analyzed by ultra-performance liquid chromatography tandem mass spectrometry (LC-MS/MS). A QC pool containing an equal mixture of samples was analyzed at the beginning and after every fourth sample (four times total). Individual samples were analyzed in a random order. Quantitative one-dimensional liquid chromatography, tandem mass spectrometry (1D-LC-MS/MS) was performed on 4 μl of the peptide digests per sample in singlicate, with additional analyses of conditioning runs and QC pools. Samples were analyzed using a nanoACQUITY UPLC system (Waters) coupled to a QExactive Plus high-resolution accurate mass tandem mass spectrometer (Thermo Fisher Scientific) via a nanoelectrospray ionization source. Briefly, the sample was first trapped on a Symmetry C18 180 μm × 20 mm trapping column (5 μl/min at 99.9/0.1 (v/v) H_2_O/methyl cyanide (MeCN)) followed by an analytical separation using a 1.7 μm Acquity HSS T3 C18 75 μm × 250 mm column (Waters) with a 90 min gradient of 5 to 40% MeCN with 0.1% formic acid at a flow rate of 400 nl/min and column temperature of 55 °C. Data collection on the QExactive Plus MS was performed in data-dependent acquisition mode with a 70,000 resolution (@ *m*/*z* 200) full MS scan from *m*/*z* 375 to 1600 with a target AGC value of 1e6 ions followed by 10 MS/MS scans at 17,500 resolution (@ *m*/*z* 200) at a target AGC value of 5e4 ions. A 20 s dynamic exclusion was employed. The total analysis cycle time for each sample injection was approximately 2 h. Following 11 total UPLC-MS/MS analyses (excluding conditioning runs but including three replicate QC injections), data were imported into Rosetta Elucidator v. 4 (Rosetta Biosoftware, Inc.), and analyses were aligned based on the accurate mass and retention time of detected ions (features) using PeakTeller algorithm in Elucidator. Relative peptide abundance was calculated based on area under the curve of the selected ion chromatograms of the aligned features across all runs. The MS/MS data were searched against a custom Swissprot database with *Homo sapiens* taxonomy with additional proteins, including yeast ADH1 and bovine serum albumin, as well as an equal number of reversed-sequence decoys for false discovery rate determination (40,546 total entries). Mascot Distiller and Mascot Server (Matrix Sciences) were utilized to produce fragment ion spectra and to perform the database searches. Database search parameters included fixed modification on Cys (carbamidomethyl) and variable modifications on Asn and Gln (deamidation). After individual peptide scoring using the PeptideProphet algorithm in Elucidator, the data were annotated at a 0.9% peptide false discovery rate. For annotation of peptides from BirA, an additional database search was performed against the GPM cRAP database (http://www.thegpm.org/crap/) containing BirA*, and BirA* peptides meeting a Mascot ion score >30 were manually annotated. In the first experiment, the dataset had 570,320 quantified features, and 577,011 high collision energy (peptide fragment) spectra that were subjected to database searching. Following database searching and peptide scoring using the PeptideProphet algorithm, the data were annotated at a 0.9% peptide false discovery rate, resulting in the identification of 24,798 peptides and 3147 proteins. After filtering the data to remove low-quality peptides with poor chromatographic peak shape and scaling of the data to the robust mean across all samples, a total of 24,821 peptides and 3146 proteins were quantified across all samples. Note that the expression value of each protein represents the aggregate of peptide expression values for that particular protein. Of these, 2167 proteins were quantified by two or more peptides, a criterion for higher confidence of identification and quantification. In the second experiment, following database searching and peptide scoring using the PeptideProphet algorithm, the data were annotated at a 1.0% peptide false discovery rate, resulting in the identification of 16,758 peptides 2522 proteins. An additional 71 peptides were annotated to BirA upon subsequent database searching. After filtering the data to remove low-quality peptides with poor chromatographic peak shape, and scaling of the data to the robust mean across all samples, a total of 16,789 peptides and 2516 proteins were quantified across all samples. Note that the expression value of each protein represents the aggregate of peptide expression values for that particular protein. Of these, 1701 proteins were quantified by two or more peptides, a criterion for higher confidence of identification and quantification. The dataset was normalized to BirA across the samples. Using the BirA*-normalized data, we examined the relative expression of PIP5K1A, which was the target of knockdown in three of the treatment groups. In knockdown cells 1 and 2, levels of biotinylated PIP5K1A were 50% lower than the wild type, whereas knockdown cell 3 had ~8-fold lower levels of PIP5K1A.

It is possible that differences in expression across treatment groups reflect the biotin capture efficiency or the overall expression level of BirA* fusion proteins. To account for these variables, two additional normalizations of the data were performed. First, to control for the variability in streptavidin pull-down, the data were normalized based on the mean intensity of peptides from endogenously biotinylated carboxylases: pyruvate carboxylase; propionyl-CoA carboxylase α-subunit; and β-methylcrotonyl-CoA carboxylase α-subunit (135 peptides total). Alternatively, we normalized based on the levels of BirA protein across each sample. The mean %CV across QC pools was basically unchanged after normalization to carboxylases (14% for all proteins; 7.7% for proteins quantified by two or more peptides) but slightly worse when the data were normalized to BirA (21.4% for all proteins; 16.5% for proteins quantified by two or more peptides). However, it was found that normalization to BirA* was effective in normalizing the total intensity of RAS proteins (including shared peptides) across the samples, so we used the BirA-normalized data for initial statistical analysis.

### CRISPR-Cas9 loss-of-function screen

To create the lentiviral library targeting the RAS interactomes an oligonucleotide pool (see Supplementary Data 8) synthesized by Custom Array comprised of five guide sequences^66^ targeting the 476 genes identified from the BirA* proximity-labeling screen as well as 50 ribosomal and non-targeting controls were cloned in the lentiCRISPR v2.0 vector by GIBSON assembly^67^. The library was packaged into lentiviruses^67^, with omission of ultracentrifugation step used to concentrate virus. Briefly, 293T/17 cells were grown in DMEM medium supplemented with 10% FBS to 50–60% confluency in a 10 cm plate. Cells were then transfected with a mixture of 3 μg of the BirA*-Ras^G12V^ CRISPR-Cas9 library, 3 μg of psPAX2, and 0.3 μg pVSVg using the Fugene 6 reagent following the manufacturer’s protocol, as above. After 24 h, the media were refreshed with DMEM medium supplemented with 30% FBS. Forty-eight hours later, 10 ml of lentivirus-containing media were filtered (0.45 μm filter) and the virus titer was determined by plating the cell line of interest in 96-well dishes with varying dilutions (1:3; 1:6; 1:9, and 1:16) of the virus-containing media to determine the lowest titer capable of achieving 90–100% cell death within 3 days of selection using 1 μg/ml of puromycin^68^. Virus was stored at −80 °C until required.

HEK-HT cells were stably infected with retroviruses derived from plasmids pWZL-Blast, pWZL-Blast-myc-KRAS^G12V^, -NRAS^G12V^, or -HRAS^G12V^ exactly as mentioned above. Five hundred thousand cells from these four polyclonal HEK-HT populations were seeded in triplicate onto 6-well plates and infected with the aforementioned lentivirus CRISPR library at a low multiplicity of infection (~0.3). Cells were cultured in 1 mg/ml puromycin for 7 days to select for infected cells. A portion of cells was then aliquoted for genomic DNA isolation to assess representation of the library (time point 0). Cells were then plated onto low serum (0.5% FBS) medium and passaged every 2 to 3 days to maintain 1000X representation of the library. Aliquots of cells were taken 7 (time point 1) and 21 (time point 2) days later for genomic DNA isolation. Genomic DNA from cells at all three time points was extracted, the small guide coding sequences were PCR amplified, barcoded, and sequenced by Illumina Hi-Seq^67^, where sequencing was performed in one direction (for primers see Supplementary Data 7).

Sequencing data were de-multiplexed using fastx_barcode_splitter (v0.0.13) to identify inline barcodes. The reads were aligned to the Ras_BirA reference library using bowtie (v1.0.0), allowing for 0 mismatches. Results at the small guide level were generated using edgeR (v3.12.1). The exact test method was used for significance testing, where a small guide was called significantly differentially expressed if |log FC| is ≥1 and false discovery rate is 5%. Abundance and depletion of small guide sequences were measured over time^69^. Briefly, a depletion metric was calculated for each small guide by calculating the abundance of sgRNA at initial time point 1 to time point 2 for the vector control, KRAS^G12V^, NRAS^G12V^, and HRAS^G12V^ samples. Further, a log_2_ ratio of the depletion metric was calculated for KRAS^G12V^, NRAS^G12V^, and HRAS^G12V^ samples to the vector control. The depletion metric for each of small guide was collapsed to the gene level by mean values of the three best small guide sequences out of the five sequences that was included in the library.

### Co-immunoprecipitation from cells

For co-immunoprecipitation assays of ectopic RAS with PIP5K1A, 293T cells were transiently co-transfected with pBabePuro-myc-KRAS^G12V^, -NRAS^G12V^, or -HRAS^G12V^ and either pLX304 or pLX304-PIP5K1A-V5 using the Fugene 6 reagent according to the manufacturer’s instructions (Promega Corporation). Forty-eight hours later, cells at >95% confluency were lysed in buffer containing 50 mM Tris (pH 8.0), 100 mM NaCl, 0.5 mM EDTA, 1% Triton X-100, 1 mM PMSF, and complete protease inhibitor tablet (Roche) for 30 min with end-to-end incubation at 4 °C. Protein concentration was measured using BCA kit. Fifty microliters of Dynabeads Protein G (Thermo Fisher Scientific) was incubated with the α-myc (1:1000) antibody for 30 min at room temperature. The beads were then washed twice in 1× PBST to remove unbound antibodies. The antibody–bead complex was incubated with the protein lysate for 2 h or overnight by end-to-end rotation at 4 °C. The beads were washed three times in wash buffer (50 mM Tris (pH 8.0), 100 mM NaCl, 0.5 mM EDTA, and 1% NP-40) for 5 min at room temperature. The eluate was separated from the beads by heating at 70 °C for 10 min followed by magnetic separation. One percent of the lysate fraction was loaded as an INPUT, while the remainder was loaded as CO-IP, resolved, and immunoblotted as above with α-myc and α-V5 (Cell Signaling, #13202; 1:1000) antibodies. The reverse co-immunoprecipitation was performed exactly as above as well, except that the immunoprecipitation was performed with the α-V5 antibody (1:1000). For co-immunoprecipitation assays of endogenous KRAS and PIP5K1A, lysates were prepared from SW620, H727, CFPac-1, and AsPc-1 cells, KRAS was immunoprecipitated with an α-KRAS (Santa Cruz Biotechnology, #sc-30; 1 μg) or IgG as a negative control (Santa Cruz Biotechnology, 1 μg) followed by immunoblot with α-KRAS (1:50) and α-PIP5K1A (Cell Signaling Technology, #9693, 1:1000) antibodies as described above. For co-immunoprecipitation assay with KRAS mutants, the experiment was performed exactly as above with the exception that 293T cells were transiently co-transfected with pBabePuro-myc-KRAS^G12V^, pBabePuro-myc-KRAS^G12V^-HHVR, or pBabePuro-myc-KRAS^G12V^-7A and pLX304-PIP5K1A-V5.

### Recombinant His10-KRAS^G12V^ and His10-HRAS^G12V^ proteins

BL21 Rosetta cells were transformed with plasmids pET21a-His10-KRAS^G12V^ or -HRAS^G12V^. At OD_600_ ~0.6, protein expression was induced with 0.5 mM isopropyl β-d-1-thiogalactopyranoside (IPTG) at 16 °C and harvested 16 h later. Cell pellets were re-suspended in Buffer A (25 mM HEPES (pH 7.5), 200 mM NaCl, and 2 mM β-mercaptoethanol (β-ME)) supplemented with 1 mM PMSF and protease inhibitor cocktail (Roche). The cells were lysed using sonication (10 s; 30 pulses) and clarified by centrifugation. The clarified lysate was passed over Ni-NTA resin that was pre-equilibrated with Buffer B (25 mM HEPES (pH 7.5), 150 mM NaCl, 5% glycerol, 2 mM β-ME, 0.03% Triton X-100, and 1 mM PMSF) containing 5 mM imidazole. The column was washed with Buffer B with increasing concentrations of imidazole (10, 20, and 50 mM). The protein was eluted with Buffer B containing 250 mM imidazole. Protein concentration was measured using NanoDrop 1000 (Thermo Fisher Scientific). The purified protein was aliquoted, flash frozen in liquid nitrogen, and stored at −80 °C.

### Recombinant GST-PIP5K1A protein

BL21-CodonPlus (DE3) RIL cells (Agilent Technologies) were transformed with plasmid pGEX-6P2-GST-PIP5K1A. Cells were grown in LB media to OD_600_ ~0.6, protein expression was induced with 0.5 mM IPTG at 25 °C, and then harvested 16 h later. Pellets of cells were re-suspended in Buffer C (50 mM HEPES (pH 7.3), 300 mM NaCl, 5% glycerol, 0.5% Triton X-100, and 2 mM β-ME) supplemented with protease inhibitor cocktail (Roche). The cells were lysed using sonication (10 s; 30 pulses) and clarified by centrifugation. The clarified lysate was passed over Glutathione Sepharose 4B resin (GE healthcare) that was pre-equilibrated with Buffer D (25 mM HEPES (pH 7.5), 150 mM NaCl, 5% glycerol, 2 mM β-ME, 0.03% Triton X-100, and 1 mM PMSF). The column was washed with 10 column volumes of Buffer D and eluted with Buffer D containing 10 mM reduced glutathione. The protein was further dialyzed overnight in Buffer D with two buffer exchanges. The protein sample was centrifuged to remove any precipitation and quantified using nanodrop 1000 (Thermo Fisher Scientific).

### In vitro pull-down

Purified recombinant His10-KRAS^G12V^ and GST-PIP5K1A proteins were incubated at 1:5 molar ratio for 30 min at room temperature. The protein mixture was then bound to a Ni-NTA column pre-equilibrated in buffer A (25 mM HEPES (pH 7.5), 150 mM NaCl, 5% glycerol, 2 mM β-mercaptoethanol, and 0.03% Triton X-100). The column was washed in Buffer A by 5 bed volumes to eliminate unbound PIP5K1A from the column matrix. A final wash was done with Buffer A containing 50 mM imidazole. His10-KRAS^G12V^ protein was eluted in Buffer A containing 250 mM imidazole in 5 fractions. The fractions were resolved by SDS-PAGE and GST-PIP5K1A and His10-KRAS^G12V^ proteins detected by Coomassie stain and immunoblotted with α-KRAS and α-PIP5K1A antibodies, as described above.

### In vitro co-immunoprecipitation

Purified recombinant His10-KRAS^G12V^ or His10-HRAS^G12V^ (50 ng) and GST-PIP5K1A (150 ng) proteins were incubated in buffer containing 10 mM Tris-HCl, pH 8.0, 100 mM NaCl, 1% Triton X-100 and protease inhibitor tablet, and incubated with 10 μl of Dynabeads Protein G for 4 h at 4 °C with 1 μg α-KRAS, α-HRAS (Santa Cruz Biotechnology, #sc-520), or IgG (Santa Cruz Biotechnology) antibody. The immune complexes were isolated after washing the beads in the same buffer for five times at room temperature and immunoblotted with α-KRAS (1:50), α-HRAS (1:50), and α-PIP5K1A (1:1000) antibodies, as described above.

### Bioinformatics

The heat map described in Fig. 1e was generated using the JMP 13.0 software from the expression values with a *z*-score 2-fold greater than the vector and a *p* value ≥0.05 from Student’s *t* test. The Venn diagram described in Fig. 1f was manually annotated based on the aforementioned dataset. Protein interaction networks described in Supplementary Fig. 2 were generated by analyzing expression values 2-fold greater than the vector using the STRING 10.5 software with a medium confidence. For analyzing protein interaction network of specific proteins, expression values 2-fold greater than the vector and the other two RAS isoforms were selected and analyzed using the STRING 10.5 software with a medium confidence. The piecharts described in Supplementary Fig. 3 were generated using Excel software of the 474 CRISPR-Cas9-targeted genes with an average sgRNA log_2_ enrichment score of ≥+0.4 or ≥−0.4 (based on the enrichment of BRAF). The waterfall plot described in Fig. 2 was generated with Excel software of the average sgRNA log_2_ enrichment of all 474 CRISPR-Cas9-targeted genes. The graph in Fig. 3a was generated using GraphPad Prism 7 based on manually curating kinases in both the proteomic and CRISPR-Cas9 screen datasets. Crystal Violet-stained cell were imaged after which colony area percentage was calculated by ImageJ using the plugin colony area. For Fig. 4e, KRAS and HRAS interactome proteins depleted or enriched upon the loss of PIP5K1A by at least a fold-difference ≥+0.1 or ≤−0.1 were analyzed for GO Enrichment using http://geneontology.org/page/go-enrichment-analysis. Supplementary Fig. 5c–f were generated using GraphPad Prism 7 of proteins proximity labeled by BirA*-KRAS^G12V^ or BirA*-HRAS^G12V^, respectively, with an average fold-difference of ≥+0.1 or ≥−0.1 between cells transduced with a vector encoding Cas9 and no sgRNA vs. those transduced with a vector encoding Cas9 and *PIP5K1A* sgRNA. This fold-difference was benchmarked to the reduction in proximity-labeling of PIP5K1A by BirA*-KRAS^G12V^ in cells expressing *PIP5K1A* sgRNA. Supplementary Fig. 5c, d display the actual degree of biotin labeling in cells with and without PIP5K1A, and Supplementary Fig. 5e, f display the fold-differences.

### Statistics

A one-way Student's *t* test was used to calculate *p* values of the differences between Crystal Violet staining of vector control cells and each of the *PIP5K1A* sgRNA-transduced cells. A two-way analysis of variance (ANOVA) test was used to calculate *p* values of the differences in the number of viable cells between (i) DMSO-treated vector control compared to DMSO-treated sgPIP5K1A cells, (ii) DMSO-treated vector control compared to trametinib-treated vector control, and (iii) DMSO-treated *sgPIP5K1A* compared to trametinib-treated *sgPIP5K1A*. The GraphPad Prism 7 software was used for all calculations.

### Data availability

Illumina Hi-seq raw data and corresponding gene annotation from the CRISPR-Cas9 screening of the RAS interactome in HRAS^G12V^-transformed, NRAS^G12V^-transformed, and KRAS^G12V^-transformed HEK-HT cells can be accessed from the Sequence Read Archive with the accession code SRP152376. Mass spectra and corresponding annotation from the proteomics analysis of proteins identified by BioID with BirA*-KRAS^G12V^, BirA*-NRAS^G12V^, and BirA*-HRAS^G12V^ expressed in HEK-HT cells and BirA*-KRAS^G12V^ or BirA*-HRAS^G12V^ expressed in HEK-HT stably transduced with a vector encoding Cas9 and no sgRNA or one targeting *PIP5K1A* can be accessed from the MassiVE repository (https://massive.ucsd.edu/) with the accession code MSV000082583. All plasmids and all other data supporting the findings of this study are available from the corresponding author on reasonable request.

## Electronic supplementary material

Supplementary Information

Description of Additional Supplementary Files

Supplementary Data 1

Supplementary Data 2

Supplementary Data 3

Supplementary Data 4

Supplementary Data 5

Supplementary Data 6

Supplementary Data 7

Supplementary Data 8
