# Supplementary material for: Interrogating the protein interactomes of RAS isoforms identifies PIP5K1A as a KRAS-specific vulnerability
Source: Nat Commun. 2018 Sep 7;9:3646. doi: 10.1038/s41467-018-05692-6 (PMC6128905; doi:10.1038/s41467-018-05692-6)
Supplement: Supplementary file 2 — Description of Additional Supplementary Files [file 41467_2018_5692_MOESM2_ESM.pdf]

Description of Additional Supplementary Files

File Name: Supplementary Data 1
Description: This table contains any protein proximity-labeled by BirA*-KRAS^{G12V} BirA*-NRAS^{G12V}, and BirA*-HRAS^{G12V}.

File Name: Supplementary Data 2
Description: This table contains enriched (2-fold) proteins proximity-labeled by BirA*-KRAS^{G12V} BirA*-NRAS^{G12V}, and BirA*-HRAS^{G12V}.

File Name: Supplementary Data 3
Description: This table contains the GO Enrichment Classification of the BirA*-RAS^{G12V} interactome identified by BioID.

File Name: Supplementary Data 4
Description: This table contains gene enrichment scores of BirA*-RAS^{G12V} interactome genes in the CRISPR/Cas9 loss-of-function screen.

File Name: Supplementary Data 5
Description: This table contains the BirA*-KRAS^{G12V} and BirA*-HRAS^{G12V} interactomes identified by proximity-labelling in vector control and *PIP5K1A* sgRNA cells.

File Name: Supplementary Data 6
Description: This table contains the genes identified with characteristics similar to PIP5K1A.

File Name: Supplementary Data 7
Description: This table contains all the primer sequences used in this study.

File Name: Supplementary Data 8
Description: This table contains all the sgRNA sequences used to generate the CRISPR Library in this study.
