# Supplementary material for: Interrogating the protein interactomes of RAS isoforms identifies PIP5K1A as a KRAS-specific vulnerability
Source: Nat Commun. 2018 Sep 7;9:3646. doi: 10.1038/s41467-018-05692-6 (PMC6128905; doi:10.1038/s41467-018-05692-6)

Supplementary Information File

Interrogating the protein interactomes of RAS isoforms identifies PIP5K1A as a KRAS-specific vulnerability

Hema Adhikari[1] and Christopher M. Counter*[1,2]

Departments of [1]Pharmacology & Cancer Biology, and [2]Radiation Oncology, Duke University Medical Center, DUMC-3813 Durham, NC, 27713. *Corresponding author (count004@mc.duke.edu).

Supplementary_Fig.1

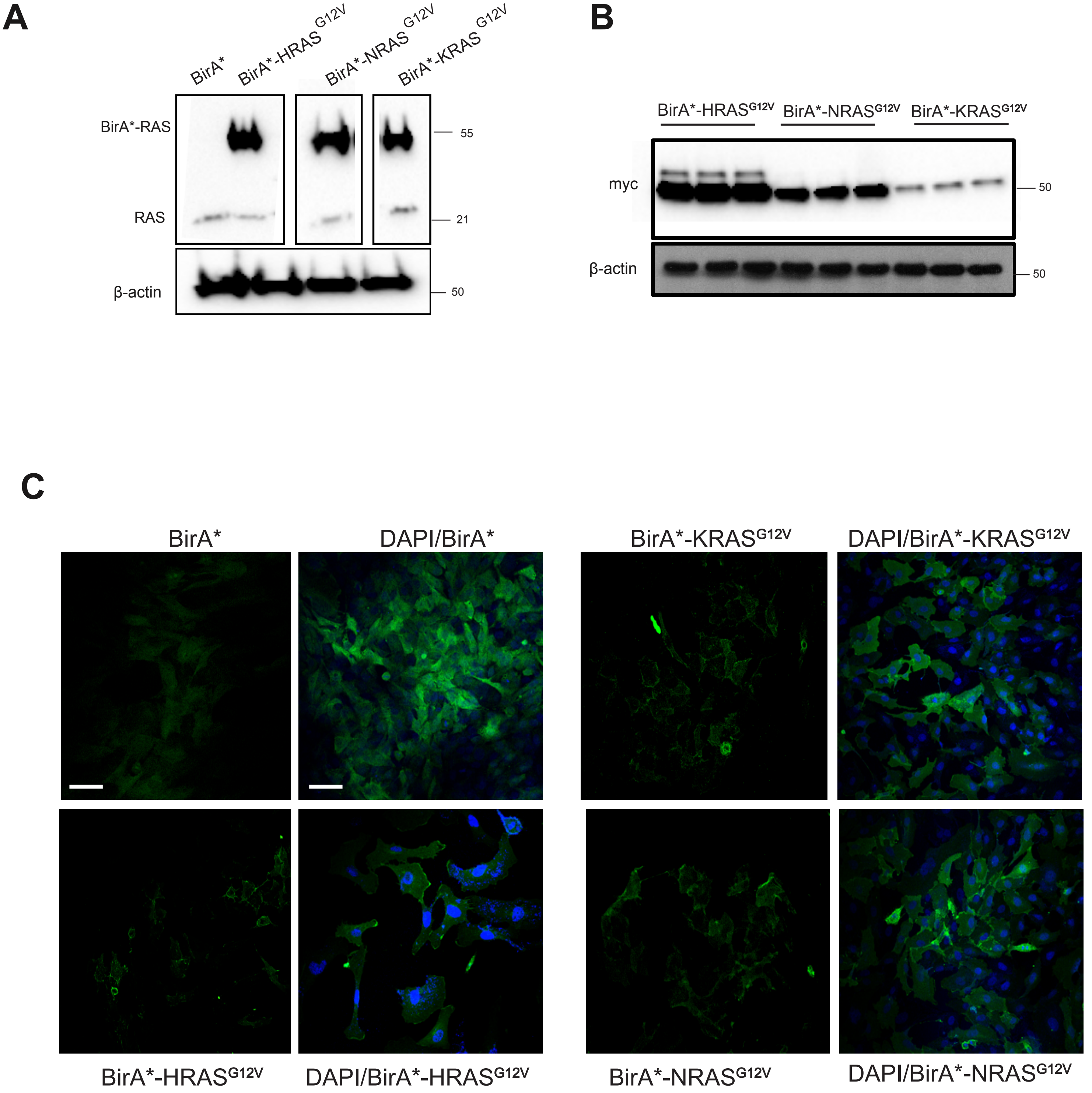

Supplementary Figure 1 | Characterizing BirA*-RAS^{G12V} expression.
(a) Comparing the protein levels of endogenous RAS to transiently expressed BirA*-RAS$^{G12V.}$ Immunoblot detection of the indicated endogenous RAS and BirA*-RAS^{G12V} proteins with an α-KRAS, α-NRAS, and α-HRAS specific antibody in HEK-HT cells transiently transduced with an expression vector encoding BirA* or BirA*-KRAS^{G12V},-NRAS^{G12V}, or -HRAS^{G12V}. β-actin serves as a loading control.
(b) Comparing protein levels of stably expressed BirA*-KRAS^{G12V},-NRAS^{G12V}, or -HRAS$^{G12V.}$ Immunoblot detection of the indicated BirA*-RAS^{G12V} proteins with an α-myc antibody in HEK-HT cells stably transduced with an expression vector encoding BirA* or BirA*-KRAS^{G12V},-NRAS^{G12V}, or -HRAS^{G12V}. β–actin serves as a loading control.
(c) Transient transfection of BirA*-RAS^{G12V} expression vectors. Representative images of the indicated myc-tagged BirA*-RAS^{G12V} proteins with an α-myc antibody by fluorescence microscopy at 20X magnification in 293T cells transiently transduced with an expression vector encoding BirA* or BirA*-KRAS^{G12V},-NRAS^{G12V}, or -HRAS^{G12V}. Scale, 10 μm

A

B

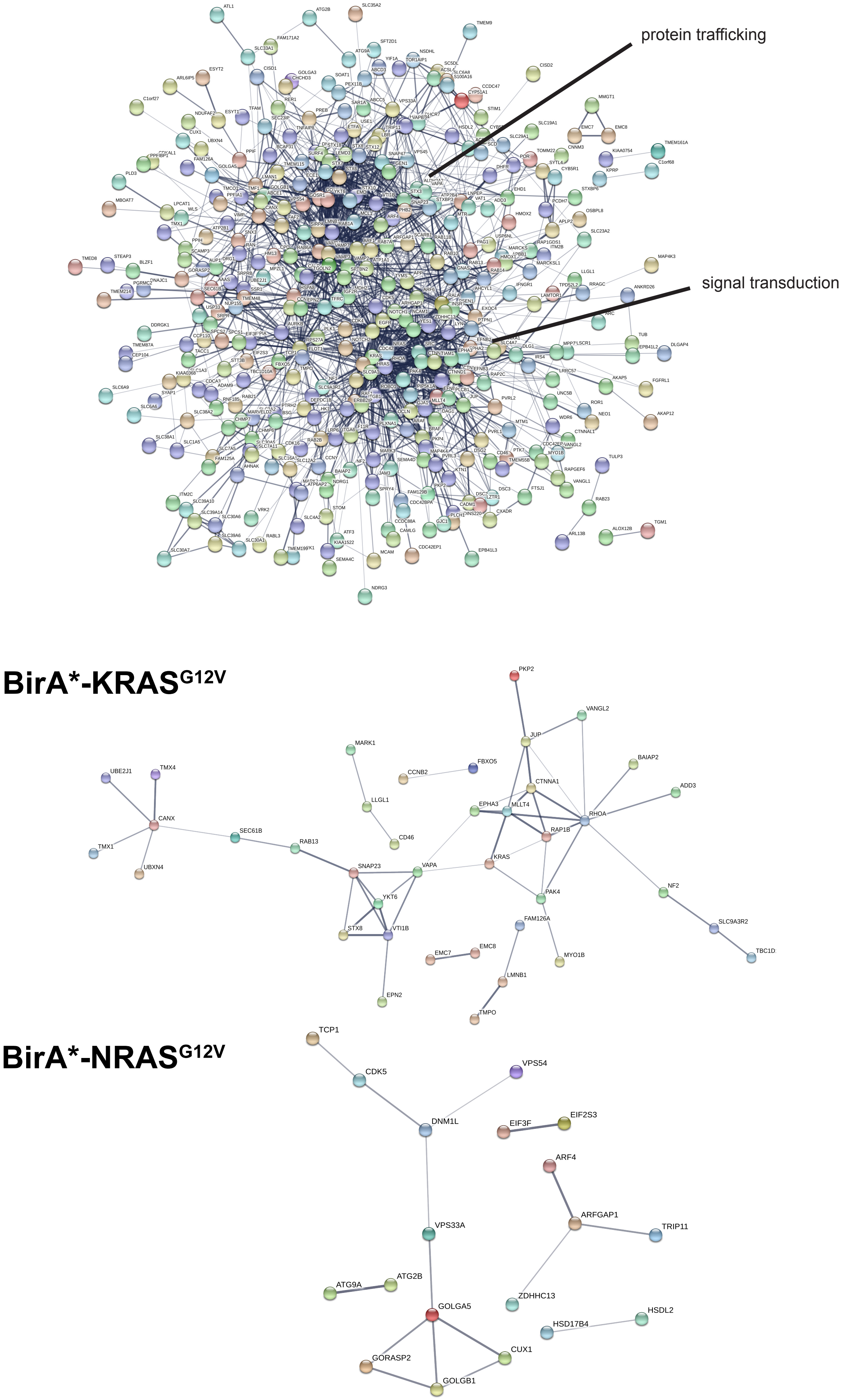

Supplementary Figure 2 | Protein network analysis of proteins proximity-labeled by BirA*-RAS. (a) Network analysis of the 477 proteins proximity-labeled by any of the three BirA*-RASG12V proteins. clusters of interactions shown. (b) Network analysis of proteins proximity-labeled by BirA*-KRASG12V (top) or -NRASG12V (bottom). Circles: clusters of interactions.

A

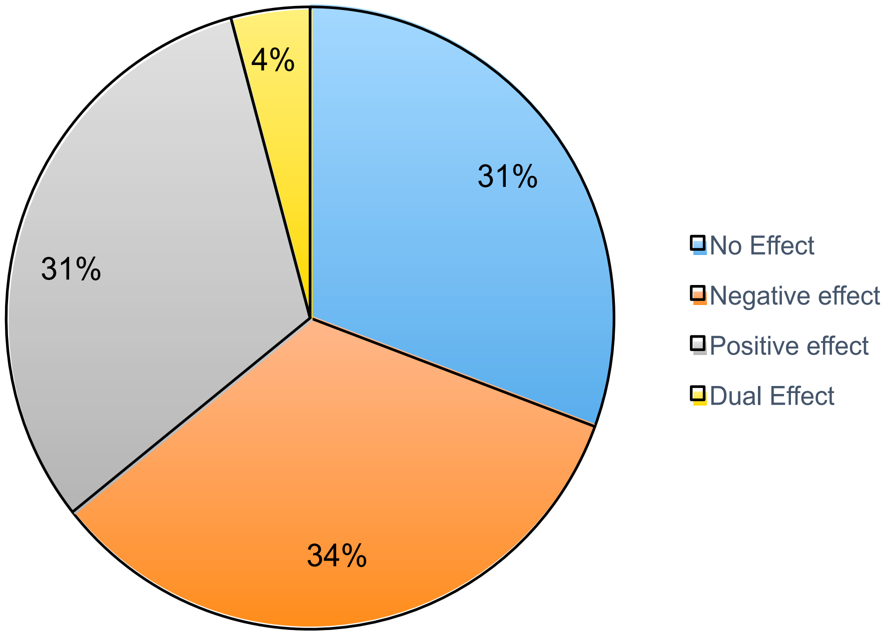

B

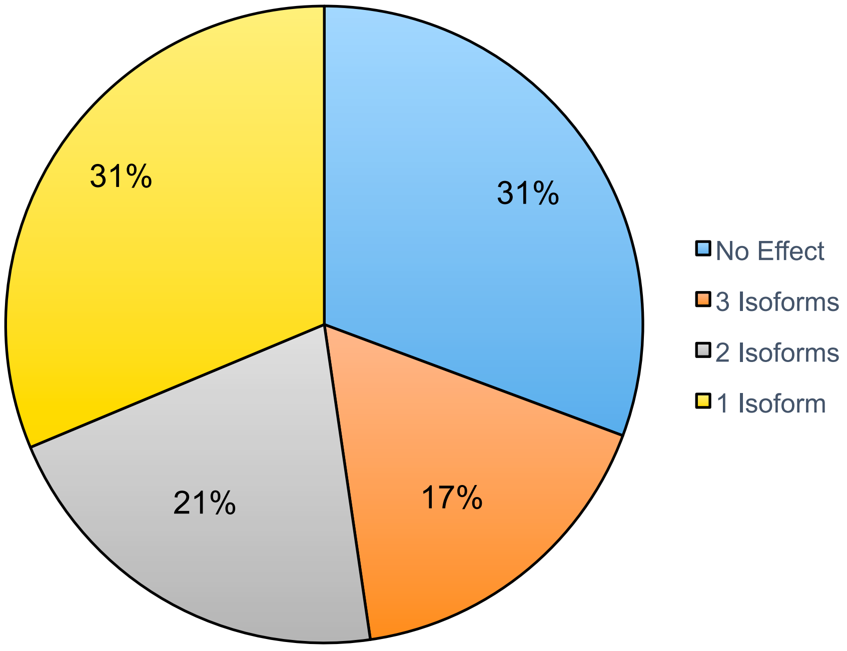

Supplementary Figure 3 | Distribution of sgRNA targeting components of the oncogenic RAS interactome defined by BioID. (a) Distribution (manually annotated) of the sgRNAs targeting the 474 genes encoding proteins identified by proximity-labeling using all three BirA*-RAS^{G12V} proteins with regards to enrichment in cells transformed by any of the three oncogenic RAS isoforms. (b) Distribution (manually annotated) of the sgRNAs targeting the 474 genes encoding proteins identified by proximity-labeling using all three BirA*-RAS^{G12V} proteins with regards to enrichment in cells transformed by none, one, two, or three oncogenic RAS isoforms.

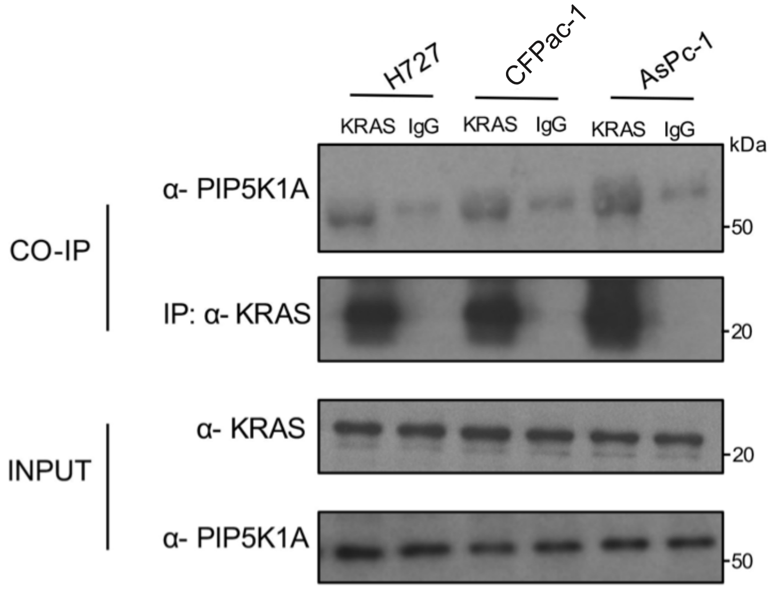

Supplementary Figure 4 | Co-immunoprecipitation of endogenous PIP5K1A with endogenous KRAS. Immunoblot detection with an α-PIP5K1A antibody of the degree PIP5K1A-V5 co-immunoprecipitates (CO-IP) with KRAS immunoprecipitated (IP) with an α-KRAS antibody or IgG as a negative control from H727, CFPac-1, and AsPc-1 cells. INPUT lysates serve to confirm PIP5K1A and KRAS expression.

Supplementary_Fig.5

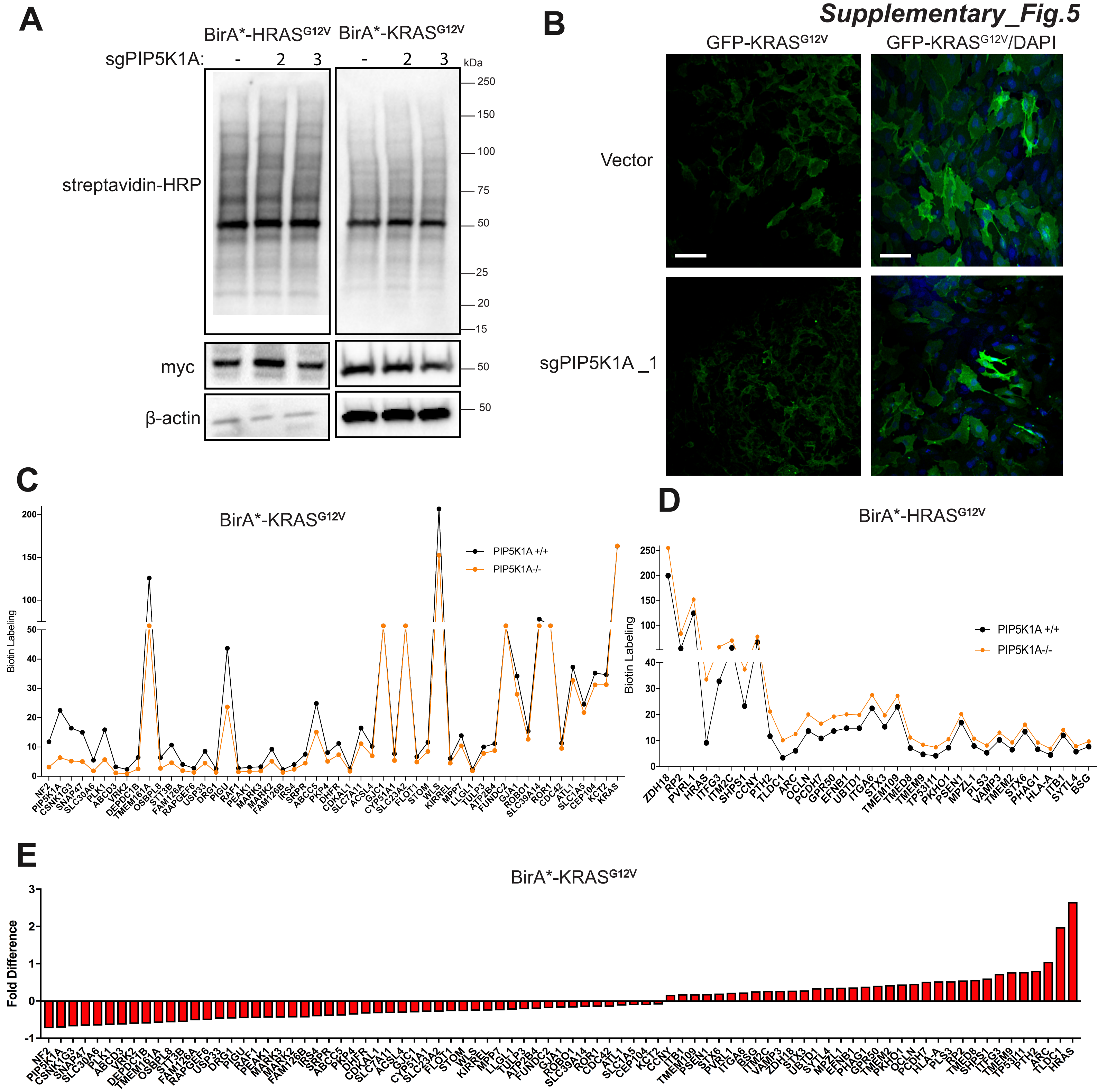

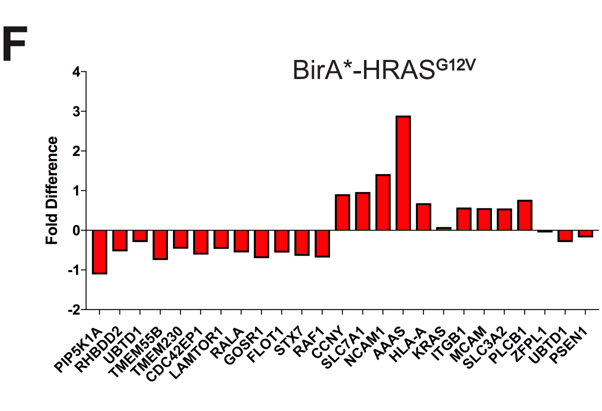

Supplementary Figure 5 | Proteins proximity-labeled by BirA*-KRASG12V versus BirA*-HRAS^{G12V} in the absence and presence of PIP5K1A. (a) Immunoblot detection of myc-tagged BirA* or the indicated BirA*-RAS^{G12V} proteins with an α-myc antibody and biotinylated proteins with HRP-streptavidin in HEK-HT cells stably transduced with a vector encoding Cas9 and no sgRNA (-) or PIP5K1A sgRNA-1 or -2 in the presence of biotin. β-actin serves as a loading control. (b) Representative images of GFP-KRAS^{G12V}, as visualized by fluorescence microscopy, when transiently transduced into 293T cells stably expressing a vector encoding Cas9 and no sgRNA (vector) or PIP5K1A sgRNA-1. Scale, 10 μm (c, d) Proximity-labeling was performed in triplicated using HEK-HT cells stably transduced with a vector encoding Cas9 and no sgRNA or one of three PIP5K1A sgRNA confirmed to reduce PIP5K1A levels (not shown) and transiently transduced with a vector encoding BirA*, BirA*-KRAS^{G12V} or BirA*-HRAS^{G12V} (36 samples). Proteins in which proximity-labeling by BirA*-KRAS^{G12V} or BirA*-HRAS^{G12V} were altered upon the loss of PIP5K1A (when benchmarked to the loss of PIP5K1A labeling by BirA*-KRAS^{G12V} in cells expressing PIP5K1A sgRNA) were identified. The average labeling in the absence and presence PIP5K1A for these proteins is plotted for (c) BirA*-KRAS^{G12V} or (d) BirA*-HRAS^{G12V}. (e,f) Fold difference of proximity-labeling of the aforementioned proteins by (e) BirA*-KRAS^{G12V} and (f) BirA*-HRAS^{G12V} in the absence versus presence of PIP5K1A.

Supplementary Fig._6

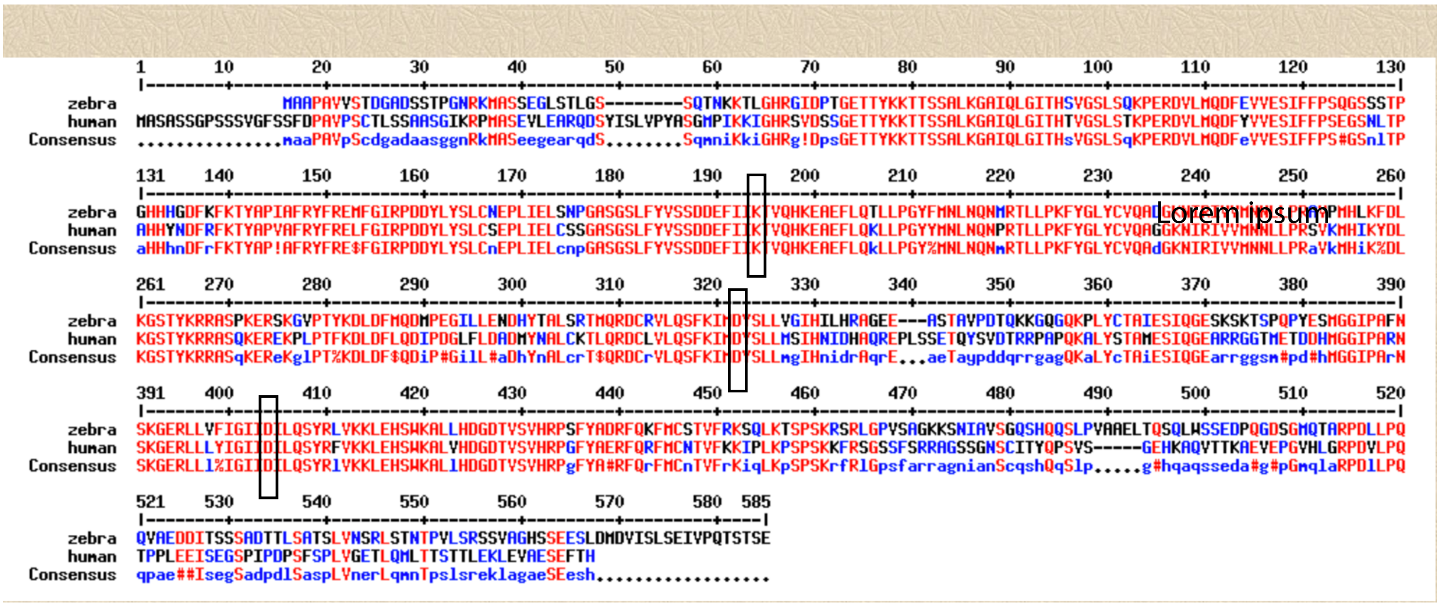

Supplementary Figure 6 | Amino acid sequence alignment of human and Zebrafish PIP5K1A. Amino acid sequence alignment of human (NCBI Gene ID: 8394) and zebrafish (NCBI Gene ID: 553629) PIP5K1A highlighting (boxed) the three sites mutated in the PIP5K1AKM mutant.

Supplementary Fig.__7

Fig.1A

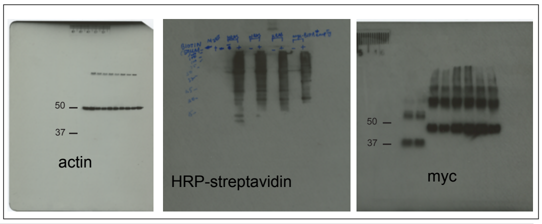

Fig.1C

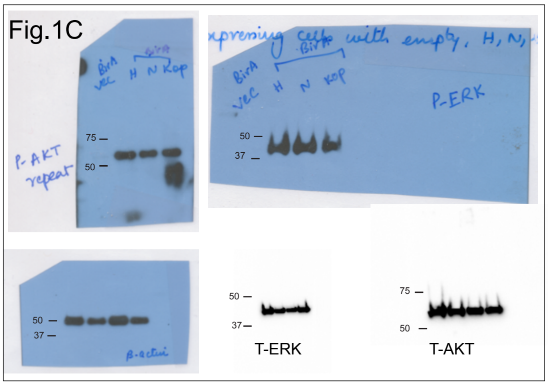

Fig.3B

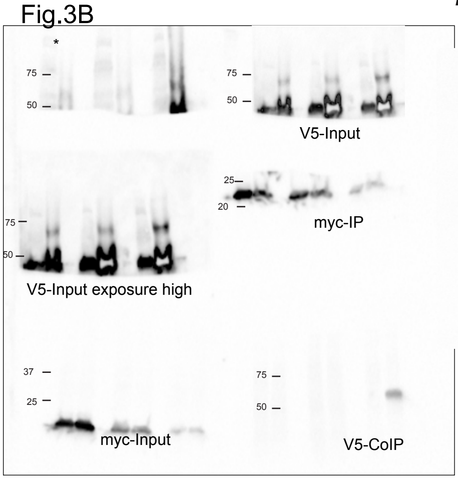

Fig.3C

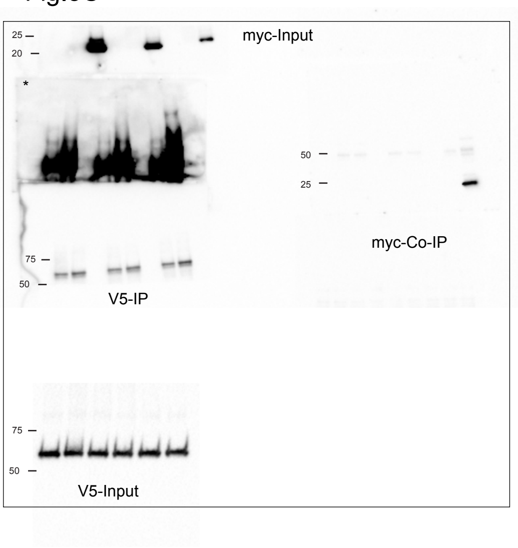

Fig.3D & S4

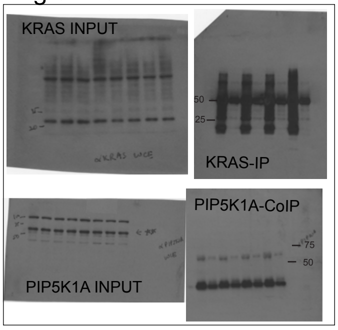

Fig.3E

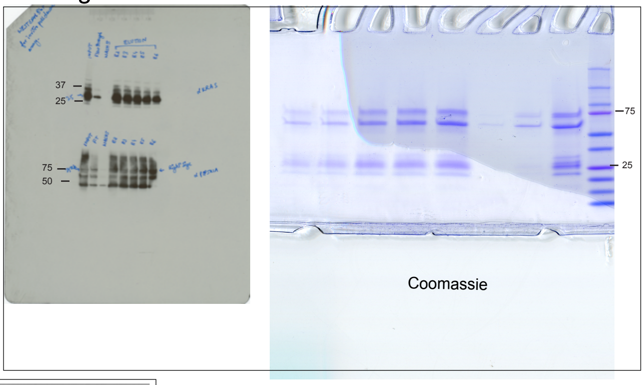

Fig.3F & 3G

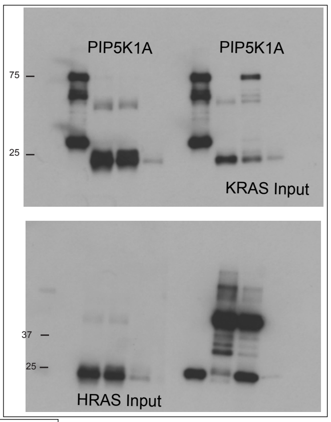

Fig.3I

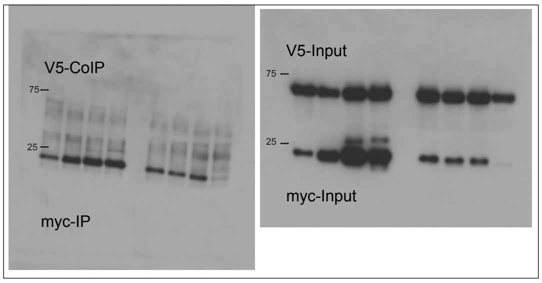

Fig.3J

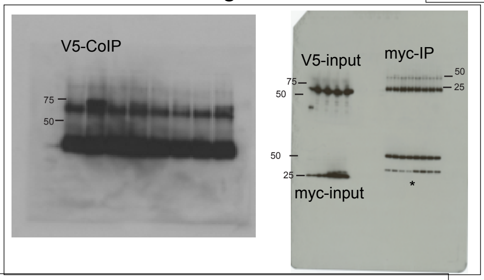

Fig.4C

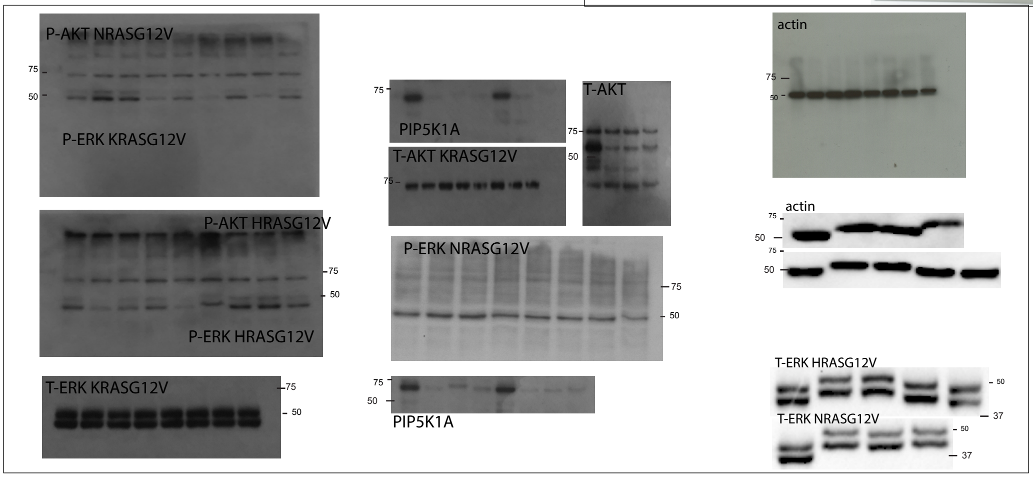

Fig.4D

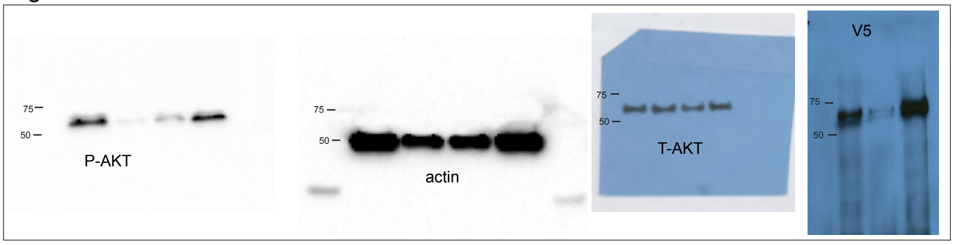

Supplementary Fig.7. Uncropped gel images

* irrlevant images

50

Supplementary_Fig.7 continued

Fig.5A

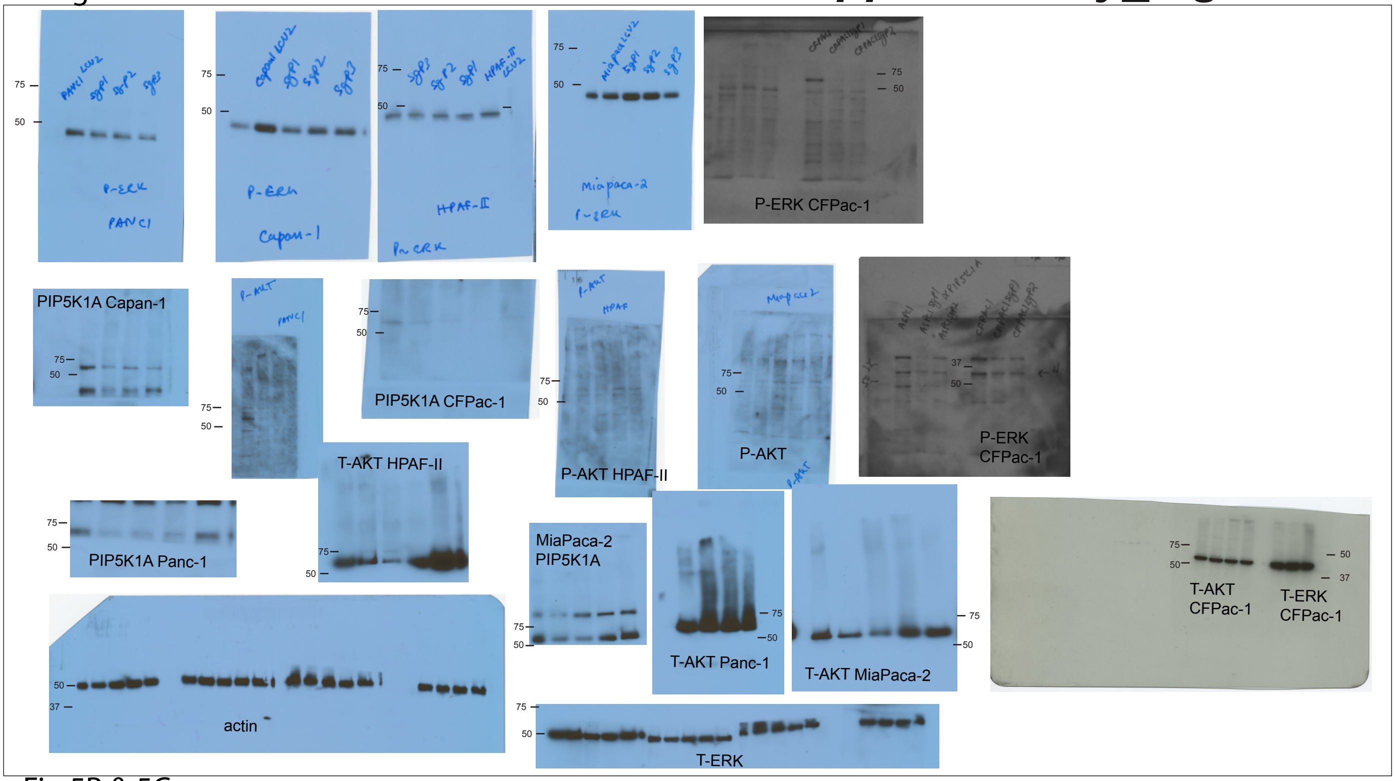

Fig.5B & 5C

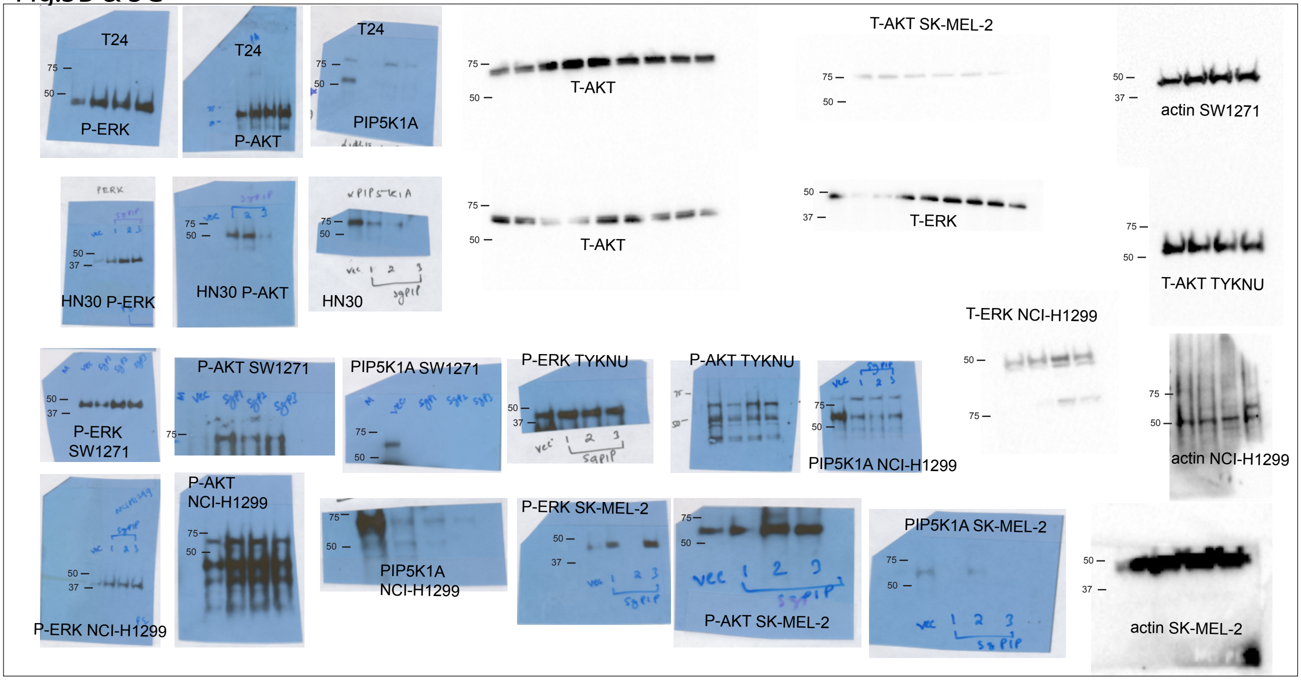

Fig.S1A

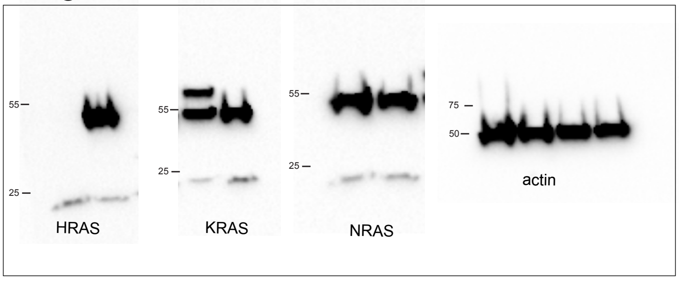

Fig.S1B

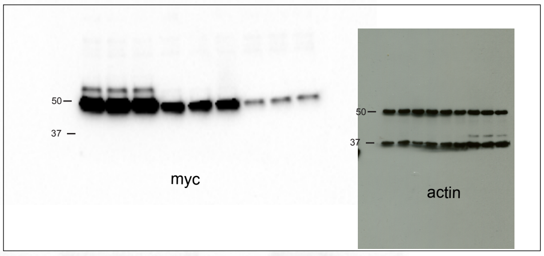

Fig.S5

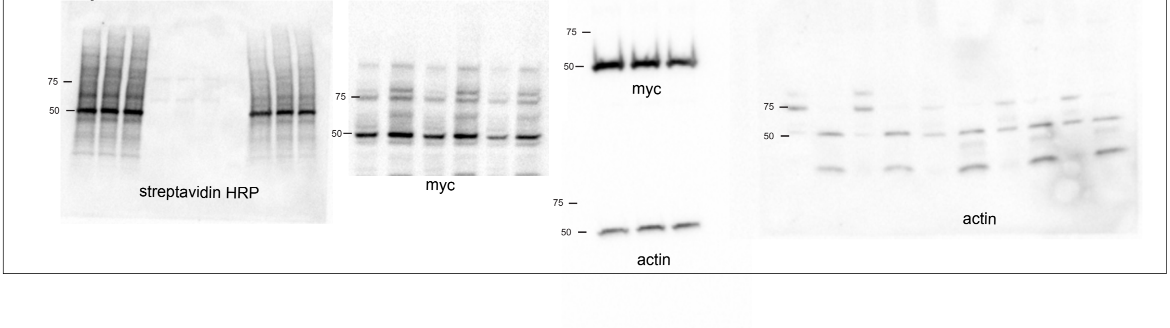

Supplement: Supplementary file 1 — Supplementary Information [file 41467_2018_5692_MOESM1_ESM.pdf]
